# A tRNA-based multiplex sgRNA expression system in zebrafish and its application to generation of transgenic *albino* fish

**DOI:** 10.1038/s41598-018-31476-5

**Published:** 2018-09-06

**Authors:** Tomoya Shiraki, Koichi Kawakami

**Affiliations:** 1 Division of Molecular and Developmental Biology, National Institute of Genetics, 1111 Yata, Mishima, Shizuoka 411-8540 Japan; 2 Department of Genetics, SOKENDAI (The Graduate University for Advanced Studies), 1111 Yata, Mishima, Shizuoka 411-8540 Japan

## Abstract

The CRISPR/Cas9 system can be introduced into zebrafish as transgenes. Namely, expression of single-guide RNA (sgRNA) and controlled expression of Cas9 in transgenic zebrafish enables the study of gene functions in specific cell types. This transgenic CRISPR/Cas9 approach would be more useful if multiple sgRNAs could be expressed simultaneously since we could knock-out a gene more efficiently or disrupt multiple genes simultaneously. Here we describe a novel system to express multiple sgRNAs efficiently in zebrafish, that relies on the endogenous tRNA processing machinery. We cloned nine endogenous zebrafish tRNA genes, fused them to sgRNAs, and demonstrated that an active sgRNA can be produced from a precursor transcript containing either of these tRNAs. To show a proof of principle, we constructed transgenic fish expressing Cas9 under the control of the *ubiquitin* promoter and a single transcript containing three distinct sgRNAs, that targeted the *slc45a2* (*albino*) gene, fused to tRNAs under the control of the U6 promoter. We found that the *Tg(ubb:SpCas9,u6c:3xslc45a2-sgRNA)* harbored mutations in all of the target sites in the *albin*o gene and showed nearly complete *albino* phenotypes, which were amenable to imaging experiments. Thus, the tRNA-based multiplex sgRNA expression system should facilitate gene knock-out studies in transgenic zebrafish.

## Introduction

The clustered regularly-interspaced short palindromic repeats (CRISPR)/Cas9 system is a bacterial adaptive immune system and has been shown to function for genome engineering in many species^1–5^. The CRISPR/Cas9 system greatly facilitates interrogation of genome functions owing to its simplicity, efficiency, and versatility. The endonuclease Cas9 is directed to genomic target sites by short RNAs, crRNA and tracrRNA, and cleaves the target sites. In the synthetically reconstituted system, these two short RNAs can be fused into a single chimeric guide RNA (sgRNA) consisting of a ~20-nt target sequence and a scaffold sequence recognized by Cas9. Co-delivery of Cas9 and sgRNA into cells enables genome editing via a non-homologous end-joining (NHEJ) or homology-directed repair (HDR) mechanism.

In zebrafish, the CRISPR/Cas9 technology allows rapid generation of knockout fish lines by simply injecting an sgRNA and the Cas9 mRNA into fertilized eggs^6,7^. Moreover, injection of multiple sgRNAs enabled multiplex biallelic genome editing in the injected zebrafish^8,9^. However, a global loss of an essential gene causes embryonic lethality or induces severe developmental defects in particular tissues, making it challenging to analyze the gene function in later stages such as in the adult. Recently, transgenic fish that expressed both sgRNAs under the control of the U6 promoter and the Cas9 gene under the control of a temporally and/or spatially controlled enhancer/promoter were generated, and a gene knock-out was carried out in a specific cell type^10–12^. A possible drawback of this method may be incomplete disruption of target genes. CRISPR/Cas9-mediated mutagenesis relies on the NHEJ repair system that usually causes small insertions or deletions (indels). Therefore, two-thirds of them result in frame-shift, and thereby only four-ninths of the target sites are expected to become biallelic frame-shift mutations. In addition, substantial cases for functional redundancy^13^ and genetic compensation^14^ (or transcriptional adaptation) have been reported in zebrafish. Therefore, it is desirable to efficiently express multiple sgRNAs that can target multiple sites in a gene or multiple genes in zebrafish.

Conventional methods employ the RNA polymerase III (Pol III)-dependent U6 promoter to express an sgRNA with defined start and end sites. One way to express multiple sgRNAs is to use multiple U6 promoters^11,12^. However, the insertion of multiple promoters at one locus in transgenic fish may cause unexpected complication in gene expression (i.e., silencing etc.). The other way to express multiple sgRNAs is to produce a single transcript containing multiple sgRNAs and process it into functional sgRNAs by a post-transcriptional processing mechanism^15–22^. In human cells, sgRNAs flanked by sequences that can be processed by Csy4, a sequence specific RNA endonuclease, has been used successfully to produce multiple sgRNAs^15,16^. The Csy4-based RNA processing however was not applied to zebrafish due to the toxicity of Csy4 in zebrafish^23^. In yeast^17^, mammalian cells^15,18^, and zebrafish^19^, hammerhead (HH) and hepatitis delta virus (HDV) ribozyme have been used to cleave RNA *in vivo* to produce multiple sgRNAs from a single transcript, which has the HH site and the HDV site on its 5′-end and 3′-end, respectively. A possible drawback of the ribozyme-mediated sgRNA generation may be reduced processing activities in accordance with increased numbers of sgRNAs^18^. Recently, the tRNA-based system utilizing the endogenous tRNA processing machinery has been described in plants, *Drosophila*, and mammalian cells^18,20–22^ and shown to produce numerous sgRNAs (at least up to eight sgRNAs)^20^. The tRNA-based system may be used to circumvent the problems associated with the ribozyme system.

In this study, we aim to develop a tRNA-based multiplex sgRNA expression system in zebrafish. We demonstrate that functional sgRNAs are efficiently produced from a single transcript containing sgRNAs flanked with zebrafish tRNAs. Furthermore, we show that this system is applicable to transgenic expression of multiple sgRNAs. Thus, the tRNA-based system should be useful to rapidly and effectively analyze gene functions in zebrafish.

## Results

### Selection of tRNA genes

**Figure 1:**
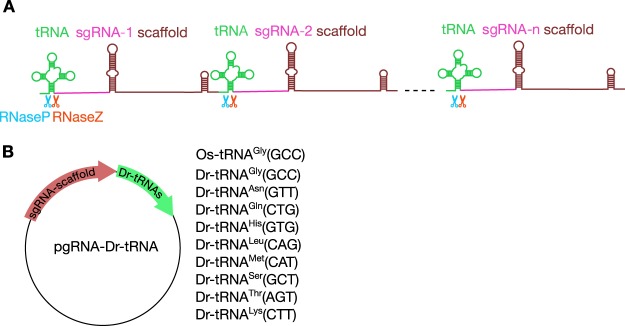
Schematic diagram of the polycistronic tRNA-sgRNA system for multiplex genome editing. (**A**) The polycistronic tRNA-sgRNA is cleaved by RNase P and RNase Z at specific sites, and each gRNA is eventually excised from the precursor transcript. (**B**) Schematic Illustration of pgRNA-Dr-tRNA plasmids used for the generation of tRNA-sgRNA transcripts. Zebrafish tRNA genes were artificially synthesized and inserted directly downstream of the sgRNA scaffold.

To develop tRNA-based multiplex sgRNA expression system (Fig. 1A), first we selected zebrafish tRNA genes that fulfill the following requirements from the GtRNAdb^24^ (Genomic tRNA Database of zebrafish genome Zv9; http://gtrnadb2009.ucsc.edu/Dreri/). Namely, we identified tRNA genes (1) whose tRNA species defined by anticodons and amino acid-based isotypes have a substantial number of genes and have the greatest gene number among their tRNA isotypes, (2) whose “Score” calculated by the tRNAscan-SE program^24^ is the highest within the tRNA species, (3) that have neither the *Bsa*I nor *Bse*RI site, both used for sgRNA cloning, and (4) that do not possess TTTT, which is the termination signal for RNA polymerase III (Pol III). Thus, we picked nine zebrafish endogenous tRNA genes (Dr-tRNA^Gly^(GCC), Dr-tRNA^Lys^(CTT), Dr-tRNA^Asn^(GTT), Dr-tRNA^Met^(CAT), Dr-tRNA^Gln^(CTG), Dr-tRNA^Ser^(GCT), Dr-tRNA^Thr^(AGT), Dr-tRNA^His^(GTG), and Dr-tRNA^Leu^(CAG)) and cloned them directly downstream of a modified sgRNA scaffold^25^ (Fig. 1B). The information of the selected tRNA genes is summarized in Supplementary Table S1.

### Activity of tRNA-sgRNA *in vivo*

**Figure 2:**
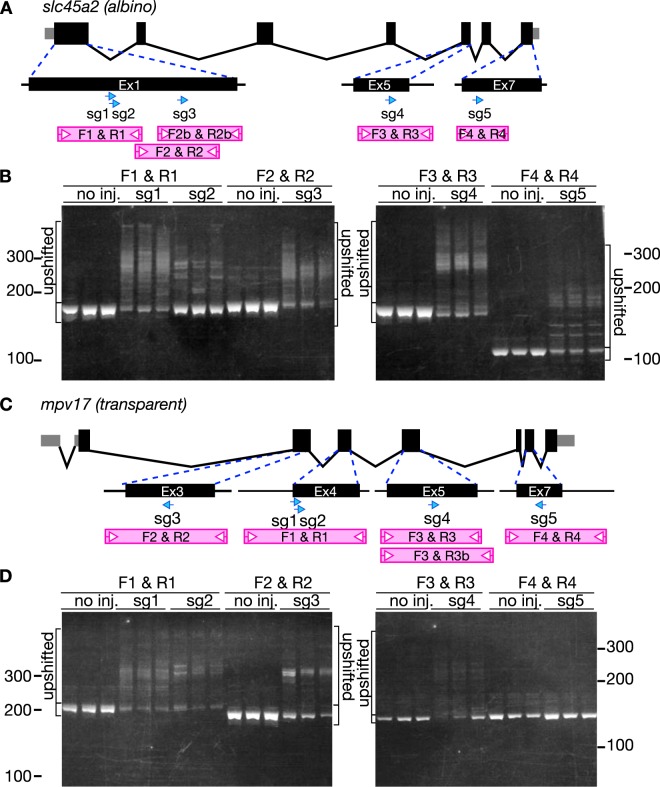
Activity of sgRNAs targeting *slc45a2* (*albino*) and *mpv17* (*transparent*). (**A**) Diagram showing the genomic structure of *slc45a2* with the five sgRNA targeting sites (blue arrows) and the PCR amplicons used for heteroduplex mobility assay (HMA) (pink boxes). Black and gray boxes indicate exons and gray zones are untranslated regions. (**B**) HMA by PAGE in uninjected embryos (no inj.) and embryos injected with a mixture containing Cas9 mRNA and each sgRNA targeting *slc45a2*. Multiple heteroduplex bands were detected in PCR amplicons from sgRNA injected embryos. (**C**) Diagram showing the genomic structure of *mpv17* with the five sgRNA targeting sites (blue arrows) and the PCR amplicons used for HMA (pink boxes). (**D**) HMA by PAGE in uninjected embryos (no inj.) and embryos injected with a mixture containing Cas9 mRNA and each sgRNA targeting *mpv17*. Multiple heteroduplex bands were detected in PCR amplicons from sgRNA-injected embryos except for sg5-injected embryos. Full gels are shown in Supplementary Fig. S7.

We designed five different sgRNAs for the *slc45a2* gene (slc45a2-sg1, -sg2, -sg3, -sg4, and -sg5) and the *mpv17* gene (mpv17-sg1, -sg2, -sg3, -sg4, and -sg5) as shown in Fig. 2A,C. *slc45a2* is the gene responsible for the zebrafish *albino* mutant^26^, and *mpv17* is the causative gene for the zebrafish *roy*/*transparent* mutant, which lacks reflective iridophores^27,28^. We synthesized these sgRNAs *in vitro*, co-injected each sgRNA with the Cas9 mRNA into fertilized eggs, and confirmed that five and four sgRNAs for *slc45a2* and *mpv17*, respectively, (slc45a2-sg1, -sg2, -sg3, -sg4, -sg5, and mpv17-sg1, -sg2, -sg3, -sg4) worked efficiently as revealed by the heteroduplex mobility assay (HMA)^29^, in which DNA heteroduplexes containing mismatches within a double-strand DNA migrate more slowly than homoduplexes (Fig. 2B,D).

**Figure 3:**
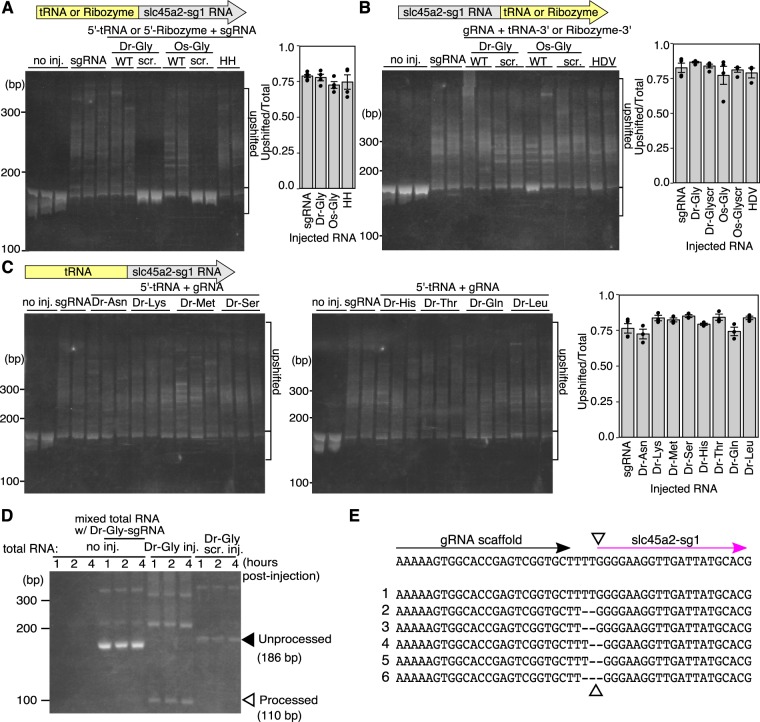
Precise excision of functional sgRNAs by the endogenous tRNA processing machinery in zebrafish embryos. (**A**) Introduction of mutations by the slc45a2-sg1 fused to tRNA at its 5′ end (5′-tRNA^Gly^(GCC)-sg1). (left) Heteroduplex mobility assay (HMA) by PAGE in uninjected embryos (no inj.), embryos injected with a mixture containing Cas9 mRNA and unfused slc45a2-sg1 (sgRNA), and embryos injected with a mixture containing Cas9 mRNA and 5′-tRNA^Gly^(GCC)-sg1. (right) Bar chart shows quantification of the ratio of heteroduplex bands. Data are shown as mean ± SEM (N = 4). Each dot represents data obtained from individual sample. Multiple heteroduplex bands were detected in PCR amplicons from wild-type tRNA-sgRNA (5′-tRNA^Gly^(GCC)-sg1) injected embryos (Dr-Gly WT and Os-Gly WT), whereas only a single band was detected in scrambled tRNA-sgRNA (5′-tRNA^Gly^(GCC)scr-sg1) injected embryos (Dr-Gly scr. and Os-Gly scr.). (**B**) (left) HMA by PAGE in uninjected embryos (no inj.), embryos injected with a mixture containing Cas9 mRNA and unfused slc45a2-sg1 (sgRNA), and embryos injected with a mixture containing Cas9 mRNA and slc45a2-sg1 fused to tRNA at its 3′-end (sg1-tRNA^Gly^(GCC)-3′). (right) A Bar chart showing the quantification of the ratio of heteroduplex bands. Data are shown as mean ± SEM (N = 3 for HDV and N = 4 for the others). Each dot represents data obtained from individual sample. Multiple heteroduplex bands were detected in PCR amplicons from both wild-type (WT) and scrambled (scr.) tRNA-sgRNAs. (**C**) (left) HMA by PAGE in uninjected embryos (no inj.), embryos injected with a mixture containing Cas9 mRNA and unfused slc45a2-sg1 (sgRNA), and embryos injected with a mixture containing Cas9 mRNA and slc45a2-sg1 fused to each of the eight species of tRNAs at its 5′-end. (right) A bar chart showing the quantification of the ratio of heteroduplex bands. Data are shown as mean ± SEM (N = 4 for sgRNA and N = 3 for tRNA-sgRNAs). Each dot represents data obtained from individual sample. Multiple heteroduplex bands were comparably detected from the embryos injected with slc45a2-sg1 fused to each of the eight tRNAs. (**D**) Detection of the mature sgRNA processed from 5′-Dr-tRNA^Gly^(GCC)-sg1 *in vivo* with cRT-PCR. The white arrowhead indicates the mature sgRNA, and the black arrowhead indicates the precursor RNA. The bands corresponding to the mature sgRNA are detected in 5′-Dr-tRNA^Gly^(GCC)-sg1 injected embryos (Dr-Gly inj.) but neither in 5′-Dr-tRNA^Gly^(GCC)scr-sg1 injected (Dr-Glyscr. inj.) nor in uninjected embryos mixed with 5′-Dr-tRNA^Gly^(GCC) -sg1 after total RNA extraction (no inj.-mixed total RNA w/Dr-Gly-sgRNA). (**E**) Sequences of mature sgRNAs that were subcloned from the fragment around 110 bp shown in D. The white arrowhead indicates the expected cleavage site. Full gels are shown in Supplementary Fig. S8.

Next, we tested whether tRNA fused to sgRNA is processed and the sgRNA produced from the tRNA-sgRNA fusion functions efficiently *in vivo*. Firstly, we constructed a fusion of the tRNA^Gly^(GCC) derived from zebrafish (Dr-tRNA^Gly^(GCC); Dr-Gly) and rice (Os-tRNA^Gly^(GCC); Os-Gly)^20^ to the slc45a2-sg1 at its 5′-end (5′-tRNA^Gly^(GCC)-sg1) or 3′-end (sg1-tRNA^Gly^(GCC)-3′). Then we injected the fused transcript synthesized *in vitro* into zebrafish embryos with Cas9 mRNA, and analyzed their cleavage activities by HMA (Fig. 3A,B). When the 5′-tRNA^Gly^(GCC)-sg1 was used for microinjection, the high cleavage activities were detected by HMA, which were comparable to injection using the sole unfused slc45a2-sg1 (Fig. 3A). In contrast, when the slc45a2-sg1 RNA flanked by a scrambled tRNA at the 5′-end (5′-tRNA^Gly^(GCC)scr-sg1) was used for microinjection with the Cas9 mRNA, no such activities were detected, indicating that the tRNA^Gly^(GCC) sequences were removed by the host tRNA processing machinery. We also injected a construct, in which the slc45a2-sg1 was fused to the hammerhead (HH) ribozyme at its 5′ end (5′-HH-sg1), and showed that the sgRNA activity was detected similarly (Fig. 3A). These indicate that the functional slc45a2-sg1 was efficiently produced from the 5′-tRNA^Gly^(GCC)-sg1 fusion transcript as well as 5′-HH-sg1. On the other hand, when sg1-tRNA^Gly^(GCC)-3′ or slc45a2-sg1 flanked by a scrambled tRNA sequence at the 3′ end (sg1-tRNA^Gly^(GCC)scr-3′) was used for microinjection with the Cas9 mRNA, both showed the activities comparable to that of the sole slc45a2-sg1 RNA, indicating that addition of ~80-nt sequences at the 3′-end of an sgRNA does not compromise its activity (Fig. 3B).

We further tested the activities of the other eight zebrafish tRNA genes (Dr-tRNA^Asn^(GTT), Dr-tRNA^Lys^(CTT), Dr-tRNA^Met^(CAT), Dr-tRNA^Ser^(GCT), Dr-tRNA^His^(GTG), Dr-tRNA^Thr^(AGT), Dr-tRNA^Gln^(CTG), and Dr-tRNA^Leu^(CAG)) by fusing them to the slc45a2-sg1 at the 5′ end, and found that all of these fusions worked well in zebrafish (Fig. 3C). We found no obvious differences in the efficiency among the nine zebrafish tRNA species, the rice Os-tRNA^Gly^(GCC), and the HH ribozyme (Fig. 3A,C).

### Processing of tRNA-sgRNA

To determine whether the tRNA was correctly processed at the molecular level, we performed circularized reverse transcription PCR (cRT-PCR) (Fig. 3D,E, Supplementary Figs S1 and S2). When the 5′-Dr-tRNA^Gly^(GCC)-sg1 was used for microinjection, a cRT-PCR product with the size of the sole slc45a2-sg1 was detected. Sequencing analysis revealed that the 5′-Dr-tRNA^Gly^(GCC)-sg1 was precisely cleaved at the junction between the tRNA and sgRNA (Fig. 3E and Supplementary Fig. S2). On the other hand, when the slc45a2-sg1 flanked by a scrambled tRNA at the 5′-end (5′-Dr-tRNA^Gly^(GCC)scr-sg1) was used for microinjection, no such products were detected, confirming that the 5′-Dr-tRNA^Gly^(GCC)-sg1 was processed dependently on the tRNA sequence (Fig. 3D). We performed a control experiment, in which the cRT-PCR reaction was carried out by using a mixture of 5′-Dr-tRNA^Gly^(GCC)-sg1 and the total RNA extracted from un-injected embryos. The cRT-PCR product was not detected, confirming the processing of the tRNA-sgRNA occurred *in vivo* (Fig. 3D). The band corresponding to the unprocessed 5′-Dr-tRNA^Gly^(GCC)-sg1 was not detectable in the injected embryos after one hour post-injection (Fig. 3D), indicating rapid and efficient tRNA-processing in zebrafish at this stage. We did not experimentally confirm the processing of tRNA fused at the 3′-end of sgRNA since the sgRNA-scrambled tRNA-3′ fusion worked efficiently (Fig. 3B).

### Activity of multiplexed sgRNAs in injected embryos

**Figure 4:**
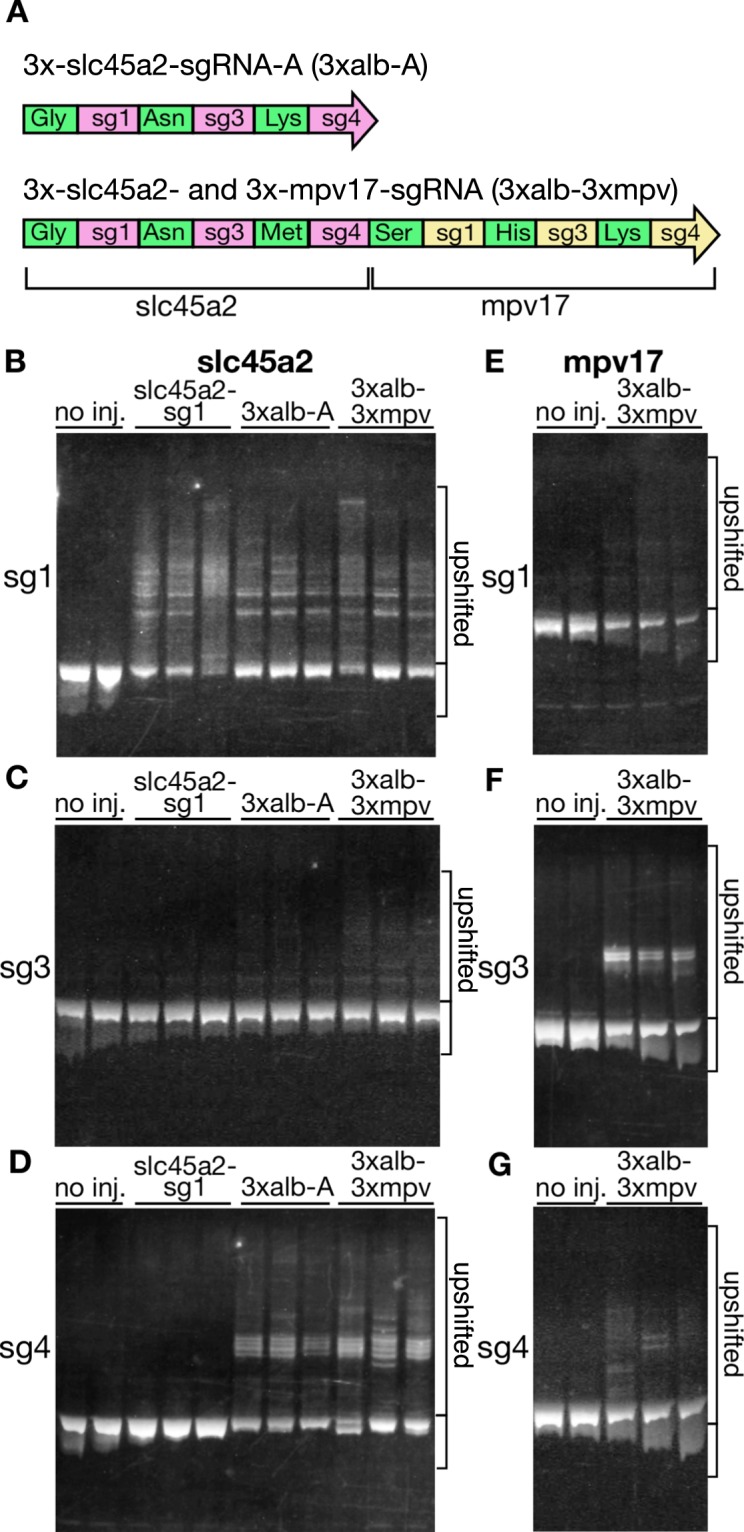
Simultaneous editing of multiple genomic sites by injecting polycistronic tRNA-sgRNAs. (**A**) Schematic depiction of injected tRNA-sgRNAs. 3xalb-A consists of an array of Dr-tRNA^Gly^(GCC), slc45a2-sg1, Dr-tRNA^Asn^(GTT), slc45a2-sg3, Dr-tRNA^Lys^(CTT), and slc45a2-sg4, while 3x-alb-3xmpv compose of an array of Dr-tRNA^Gly^(GCC), slc45a2-sg1, Dr-tRNA^Asn^(GTT), slc45a2-sg3, Dr-tRNA^Met^(CAT), slc45a2-sg4, Dr-tRNA^Ser^(GCT), mpv17-sg1, Dr-tRNA^His^(GTG), mpv17-sg3, Dr-tRNA^Lys^(CTT), and mpv17-sg4. (**B**–**G**) Detection of mutations (upshifted bands) at multiple genomic sites in embryos injected with a mixture containing Cas9 mRNA and each of the tRNA-sgRNAs. Heteroduplex mobility assay (HMA) of PCR amplicons surrounding the target sequences of slc45a2-sg1 (**B**), slc45a2-sg3 (**C**), slc45a2-sg4 (**D**), mpv17-sg1 (**E**), mpv17-sg3 (**F**), mpv17-sg4 (**G**). Full gels are shown in Supplementary Fig. S9.

To determine whether multiple functional sgRNAs can be produced from a single precursor transcript containing an array of tRNA-sgRNAs, precursor transcripts, 3xalb-A (Dr-tRNA^Gly^(GCC)-slc45a2-sg1-Dr-tRNA^Asn^(GTT)-slc45a2-sg3-Dr-tRNA^Lys^(CTT)-slc45a2-sg4) containing three distinct sgRNAs against the *slc45a2* gene and 3xalb-3xmpv (Dr-tRNA^Gly^(GCC)-slc45a2-sg1-Dr-tRNA^Asn^(GTT)-slc45a2-sg3- Dr-tRNA^Met^(CAT)-slc45a2-sg4- Dr-tRNA^Ser^(GCT)-mpv17-sg1- Dr-tRNA^His^(GTG)-mpv17-sg3- Dr-tRNA^Lys^(CTT)-mpv17-sg4) containing six distinct sgRNAs against the *slc45a2* and *mpv17* genes, were synthesized *in vitro* and injected into zebrafish embryos with the Cas9 mRNA (Fig. 4). When the 3xalb-A (targeting *slc45a2*) was used for microinjection, the cleavage activities of the three sgRNA were detected by HMA, though the activity of slc45a2-sg3 was weakly detected (Fig. 4B–D). Also, when the 3xalb-3xmpv (targeting *slc45a2* and *mpv17*) was used for microinjection, multiple heteroduplex bands were observed in all of the six target regions by HMA (Fig. 4B–G).

**Figure 5:**
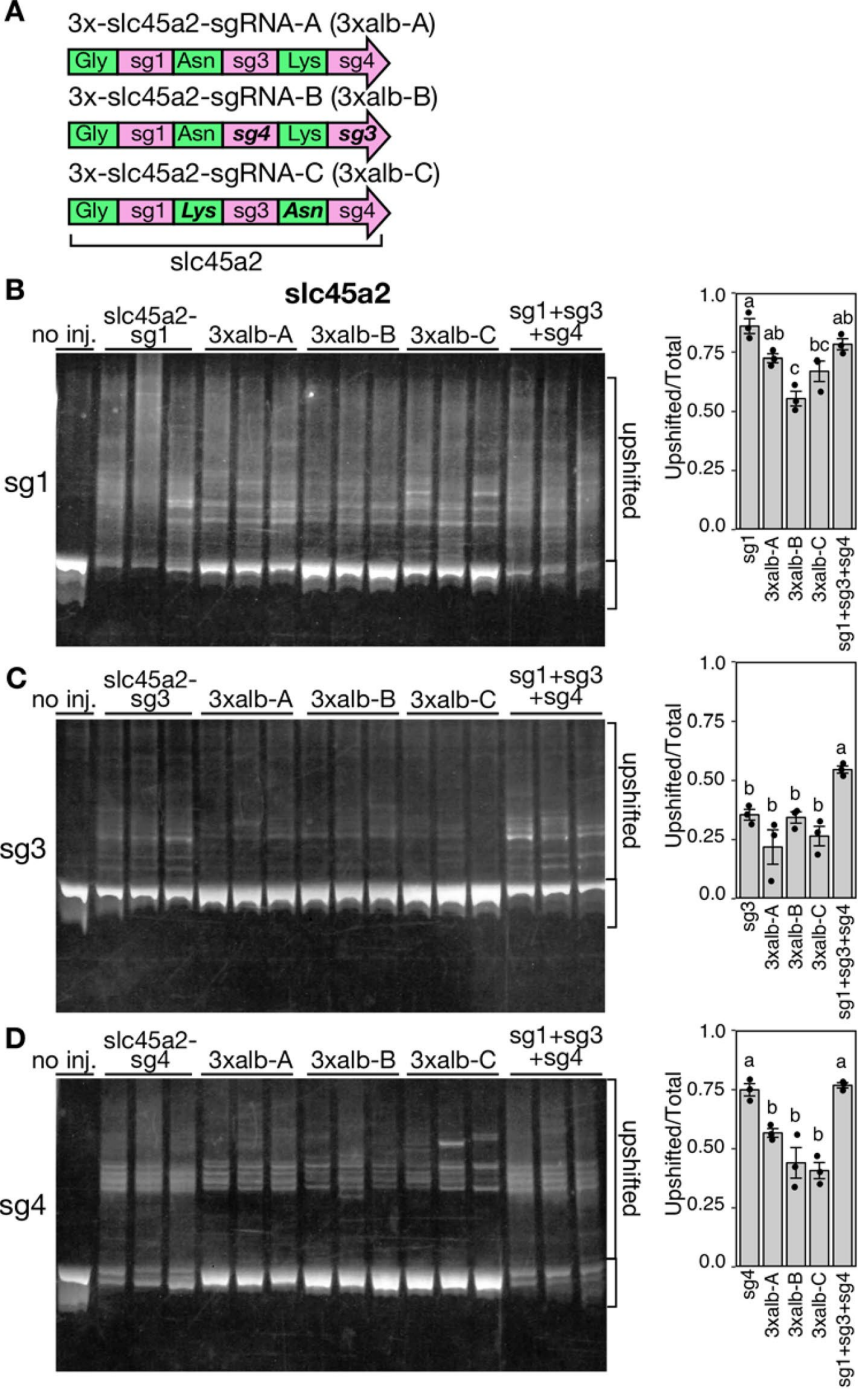
Comparison of the activities of 3xalb tRNA-sgRNAs carrying tRNAs and sgRNAs in different orders. (**A**) Schematic depiction of 3xalb tRNA-sgRNAs. 3 × -slc45a2-sgRNA-A (3xalb-A), the original; 3 × -slc45a2-sgRNA-B (3xalb-B); sg3 and sg4 were switched (bold italic); 3 × -slc45a2-sgRNA-C (3xalb-C), Asn and Lys tRNAs were switched (bold italic). (**B**–**D**) (left) Heteroduplex mobility assay (HMA) of PCR amplicons amplified from the target sequences of sg1 (**B**), sg3 (**C**), and sg4 (**D**) in embryos injected with a mixture containing Cas9 mRNA and each 3xalb tRNA-sgRNA. Embryos injected with single sgRNAs synthesized *in vitro* (slc45a2-sg1 in B, slc45a2-sg3 in C, and slc45a2-sg4 in D) and embryos simultaneously injected with three sgRNAs (sg1 + sg3 + sg4) were also analyzed. (right) Bar charts show quantification of the ratio of the heteroduplex bands. Data are shown as mean ± SEM (N = 3). Dots represent data obtained from individual samples. Means with the same letter are not significantly different (*P* < 0.05, Tukey test). Full gels are shown in Supplementary Fig. S10.

The 3xalb-A tRNA-sgRNAs showed lower activities at the sg3 site compared to those of the sg1 and sg4 sites (Figs 2B and 4C). To examine the possibility that the position of sgRNAs and/or the flanking tRNA species in 3xalb tRNA-sgRNAs affect the efficiency of tRNA processing, we synthesized two additional versions of 3xalb-sgRNAs, 3xalb-B (Dr-tRNA^Gly^(GCC)-slc45a2-sg1-Dr-tRNA^Asn^(GTT)-slc45a2-sg4-Dr-tRNA^Lys^(CTT)-slc45a2-sg3) and 3xalb-C (Dr-tRNA^Gly^(GCC)-slc45a2-sg1-Dr-tRNA^Lys^(CTT)-slc45a2-sg3-Dr- tRNA^Asn^(GTT)-slc45a2-sg4), in which the order of sgRNAs (3xalb-B) and tRNAs (3xalb-C) was changed from the original one (3xalb-A), respectively (Fig. 5A). We injected these three tRNA-sgRNAs into zebrafish embryos with the Cas9 mRNA, and examined their activities by HMA (Fig. 5B–D). The HMA analyses detected no significant differences in the activities at the sg3 and sg4 sites (Fig. 5C,D), indicating that the positions of sgRNAs or the flanking tRNA species does not affect the Cas9 cleavage activity at the target site.

Simultaneous expression of multiple sgRNAs (tRNA-sgRNAs) may cause unwanted competition between them. To test this, embryos were injected with a mixture of three sgRNAs (sg1 + sg3 + sg4 in Fig. 5), and the activities were compared to those in embryos injected with single sgRNAs. As shown in Fig. 5B–D, co-injection of three sgRNAs did not reduce the Cas9 activities at the target sites. It is possible that, if the sg1 and sg4 sites were cleaved efficiently, the genomic region between them could be deleted^30^ and the sg3 site could be lost. The activities at the sg3 site were not decreased (and even higher) in embryos injected with all three sgRNAs, suggesting such a possible genomic deletion does not affect this assay system. On the other hand, we detected lower activities of sg3 when 10 ng/µL gRNA was used for injection (Fig. 5C) than sg1 and sg4, instead of 25 ng/µL used in Fig. 2B, and this lower activity of sg3 may attribute to the lower activities of sg3 in Fig. 4C. Taken together, our results demonstrated that the endogenous tRNA processing system could efficiently produce multiple (at least up to six) functional sgRNAs from a single precursor transcript in zebrafish.

### Generation of transgenic *albino* zebrafish

**Figure 6:**
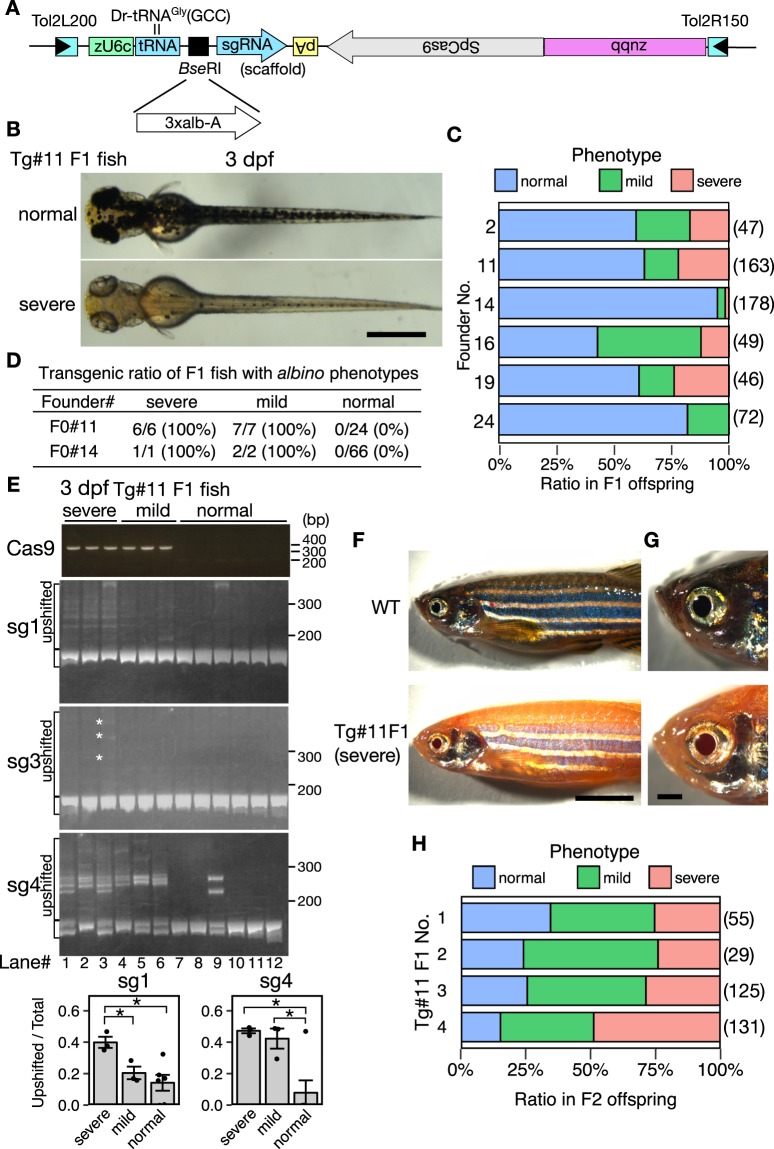
Generation of transgenic *albino* zebrafish by using tRNA-based multiplexed sgRNA expression. (**A**) Structure of DNA construct used for the generation of transgenic *albino* zebrafish. Three distinct sgRNAs targeting the *albino* (*slc45a2*) locus were cloned at the *Bse*RI site of an all-in-one *Tol2* vector, pT2TS-ubb:Cas9;u6c:Dr-tRNA^Gly^(GCC)-sgRNA-scaffold, and are transcribed as a single polycistronic RNA under the zebrafish *u6c* promoter. Cas9 is driven by the *ubb* promoter, and its direction is opposite to that of the u6c:sgRNAs. (**B**) Representative images of the dorsal view of 3-dpf larvae derived from a founder fish (#11). Some larvae show severe *albino* phenotype (lower), whereas most larvae show normal pigmentation (upper). (**C**) The rate of F1 larvae showing *albino* phenotype at 3 dpf for each founder fish. Numbers in parentheses indicate the number of F1 larvae examined. (**D**) Transgenic ratio of F1 fish with *albino* phenotypes derived from two founders, F0#11 and F0#14. (**E**) Detection of mutations at multiple genomic sites of the *slc45a2* locus in transgenic-albino larvae. (top) Genotyping of transgenic fish was performed by PCR using primers on SpCas9 genes. (middle three panels) Detection of mutations at multiple genomic sites of the *slc45a2* locus by Heteroduplex mobility assay (HMA). Mutations were introduced in all the three targeting sites in a transgenic larva (lane#3) that showed severe *albino* phenotype. White asterisks on the HMA gel image of sg3 denote faint heteroduplex bands. (bottom) Bar charts show quantification of the ratio of the heteroduplex bands in sg1 and sg4. Data are shown as mean ± SEM (N = 3 for sever and mild, and N = 6 for normal). Dots represent data obtained from individual samples. Asterisks indicate significant differences (*P* < 0.05, Student’s t test). (**F**) Representative images of the lateral views of adult wild-type (WT; upper) and transgenic-albino (Tg; lower) fish. (**G**) Magnified images of the head region of the fish shown in F. (H) The rate and severity of the *albino* phenotype in F2 offspring at 3 dpf produced from four F1 fish derived from Tg#11. Numbers in parentheses indicate the number of F2 larvae examined. Scale bars: 500 µm (**B**), 5 mm (**F**), 1 mm (**G**). Full gels are shown in Supplementary Fig. S11.

To demonstrate that the tRNA-based system is applicable to transgenic expression of multiple sgRNAs in zebrafish, we created transgenic zebrafish carrying the 3xalb-A (Dr-tRNA^Gly^(GCC)-slc45a2-sg1-Dr-tRNA^Asn^(GTT)-slc45a2-sg3-Dr-tRNA^Lys^(CTT)-slc45a2-sg4). In this experiment, at first we constructed an all-in-one *Tol2* vector pT2TS-ubb:Cas9;u6c:Dr-tRNA^Gly^(GCC)-sgRNA-scaffold, that contains the zebrafish U6c promoter^11^, zebrafish tRNA^Gly^(GCC), a cloning site (*Bsa*I and *Bse*RI) for multiple tRNA-sgRNAs, a modified sgRNA scaffold^25^, and the Cas9 gene under the control of the zebrafish *ubiquitin b* promoter^31^ (see Materials and Methods; Fig. 6A and Supplementary Fig. S6). We then constructed pT2TS-ubb:Cas9;u6c:3xalb-A, in which 3xalb-A (Fig. 4A) was cloned into the *Bse*RI site of pT2TS-ubb:Cas9;u6c:Dr-tRNA^Gly^(GCC)-sgRNA-scaffold (Fig. 6A).

Zebrafish embryos injected with pT2TS-ubb:Cas9;u6c:3xalb-A were raised to adulthood to establish stable transgenic lines. We found that 20–40% of the injected fish (F0) showed mosaic *albino* phenotypes at adult stage (Supplementary Fig. S3A). Of 20 F0 fish examined, we obtained six founder fish that transmitted the transgene to the F1 generation with sufficiently high frequencies (>10%) by PCR genotyping in which a small number of embryos (~5) were pooled (data not shown). At 3 dpf, F1 larvae derived from all these six founders showed mosaic *albino* phenotypes, either severe or mild phenotypes, with varied frequencies (Fig. 6B,C and Supplementary Fig. 3B). These frequencies (Fig. 6C) were approximately matched with the estimated germ-line transmission frequencies (data not shown), suggesting that the F1 transgenic larva had the *albino* phenotype presumably due to biallelic disruption of the *slc45a2* gene. To confirm this, we analyzed the individual F1 larvae from two founder fish, #11 and #14, by PCR-based genotyping at 5 dpf. The larvae showing severe and mild *albino* phenotypes always carried the transgene while normally-pigmented larvae did not (Fig. 6D and Supplementary Fig. S4). Some of the F1 larvae showed a nearly complete *albino* phenotype similarly to the *albino* mutant^26^ (Fig. 6B and Supplementary Fig. S3B). In these larvae, melanophores in both the body and retinal pigment epithelium (RPE) failed to gain pigmentation properly at 3 dpf, and the lack of melanization persisted to adulthood (Fig. 6F,G and Supplementary Fig. S3C,D).

To test whether mutations at multiple target sites were introduced in the Tg-albino fish, we analyzed the normal, severe and mild *albino* F1 larva by HMA (Fig. 6E). Consistent with the RNA injection experiments (Fig. 4B,D), the HMA bands, that were indicative of mutations at the target sites in somatic cells, were well detected at the both sg1 and sg4 sites. The activity at the sg3 site was weaker, as observed in the RNA injection experiment (Fig. 4C), but the HMA bands could be detected at least one F1 larva with severe *albino* phenotype (lane 3 in Fig. 6E). These indicated that three functional sgRNAs (slc45a2-sg1, slc45a2-sg3, and slc45a2-sg4) were produced in the transgenic fish. Then we compared the HMA bands in the larvae with the severe and mild *albino* phenotypes. The larvae with the severe *albino* phenotype contained more mutations than the mild *albino* phenotype, especially at the sg1 site (lanes 1–6 in Fig. 6E), supporting the notion that expression of multiple sgRNAs should facilitate generation of severer phenotypes.

We further crossed the Tg-albino F1 fish from the founder #11 with wild type (WT) fish, and analyzed the phenotypes of the F2 offspring. The Tg-albino F2 larvae also showed severe and mild *albino* phenotypes at the frequencies of more than 50% (Fig. 6H), which were likely to be due to multi-copy integrations in the parental F1 fish (No. 1–4 in Fig. 6H). We analyzed the F2 fish with severe *albino* phenotypes by Southern blot hybridization and confirmed that they still had at least three copies of the transgenes (data not shown). These results demonstrated that the transgenic fish carrying the all-in-one pT2TS-ubb:Cas9;u6c:3xalb-A construct in the hemizygous condition could generate *de novo* homozygous *albino* mutations in somatic cells (Fig. 6 and Supplementary Fig. S3).

### Fluorescence imaging in the head of Tg-albino

Zebrafish is highly suited for *in vivo* imaging owing to its transparent body at early embryonic stages. However, at larval stages the optical accessibility is significantly lowered by development of melanophores. Previous studies employed chemical compounds 1-Phenyl-2-thiourea (PTU)^32^, or mutations, such as *nacre*, *casper* and *albino*^33–35^, to reduce such unwanted pigmentation. Our results suggested that the Tg-albino fish may be used as a new way to circumvent the problem.

**Figure 7:**
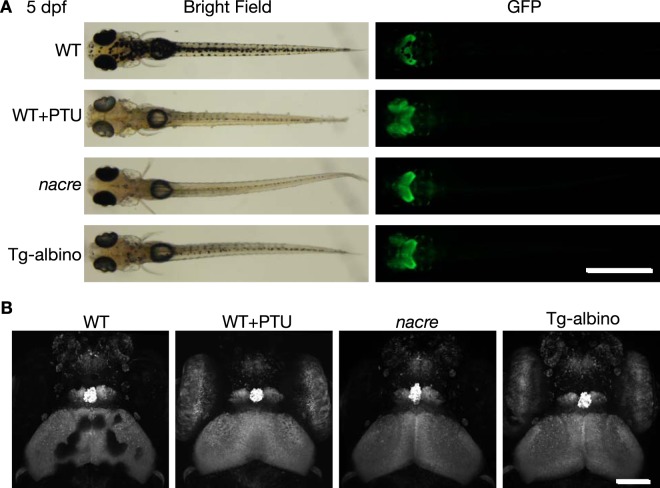
Fluorescent images of *Gt(gSA2AzGFF49A);Tg(UAS:GFP)* at 5 dpf in Tg-albino background. (**A**) Representative bright field (left) and fluorescent (GFP; right) images of dorsal views of *Gt(gSA2AzGFF49A);Tg(UAS:GFP)* in wild-type (WT), PTU-treated WT (WT + PTU), *nacre*, and Tg-albino backgrounds. (**B**) Two-photon imaging of head regions in *Gt(gSA2AzGFF49A);Tg(UAS:GFP)*. Images are displayed as a maximum projection of z-stacks. Scale bars: 1 mm (**A**), 100 µm (**B**).

We tested if the Tg-albino larvae are transparent enough for imaging analysis. Bright field images showed that the Tg-albino F2 larvae had severe *albino* phenotypes in the body and eyes, and were comparable to the *albino* homozygous mutant and PTU (1-Phenyl-2-thiourea)-treated larvae (Supplementary Fig. S5). Then, the Tg-albino F1 fish was crossed with *Gt(gSA2AzGFF49A);Tg(UAS:GFP)* (49A-gal4;UAS:GFP), which expresses GFP in the optic tectum, habenula, pineal gland, and eye^36^. Fluorescence images of the 49A-gal4;UAS:GFP larvae in the Tg-albino background were acquired and compared to those of the 49A-gal4;UAS:GFP, PTU-treated 49A-gal4;UAS:GFP, and *nacre*;49A-gal4;UAS:GFP larvae (Fig. 7). GFP expression in the brain area of Tg-albino could be observed as well as the *nacre* and PTU-treated fish, whereas in the 49A-gal4;UAS:GFP larvae, the GFP signal was interrupted by the dark pigments (Fig. 7). *nacre* lacks all melanophores due to a mutation in the *mitfa* gene^34^, but still retains pigmented eyes. In contrast, the Tg-albino fish lacked melanin not only in the melanophores but also in the RPE, and therefore GFP expression in the eye can be observed similarly to the PTU-treated fish (Fig. 7). Importantly, unlike PTU-treated fish that showed delayed development, the Tg-albino fish did not show such growth retardation (Fig. 7). Collectively, our data demonstrated that the Tg-albino fish should be useful for *in vivo* imaging and could be an attractive alternative to PTU treatment or the *nacre* mutation.

## Discussion

In this study, we established the tRNA-based multiplex sgRNA expression system in zebrafish. Previous studies that reported tRNA-based multiplex sgRNA expression system in rice, *Drosophila* and mammalian cells employed one type of tRNAs, tRNA^Gly^(GCC), derived from rice or *Drosophila*^18,20–22^. In contrast, we cloned nine tRNA genes from zebrafish and demonstrated that all of the nine tRNAs can be processed *in vivo* in zebrafish when they are fused with sgRNA. We think that the use of the endogenous tRNA genes from zebrafish may have the following merits. First, the tRNA genes (~80-nt) are shorter than the ribozymes (HH + HDV; ~120-nt), and are varied in sequences. Thus, we can have more choices for the tRNA sequence and avoid to use a same tRNA sequence repeatedly when an array of multiple gRNA-tRNA sequences is created. This feature could reduce instability or silencing effects possibly caused by tandem repeats, and therefore would lead to reliable transgene expression. Second, compared to the other methods employing ribozymes^19^ and Csy4 ribonuclease^23^, the latter reported as toxic in zebrafish, the endogenous tRNA processing system should be less toxic in zebrafish. In fact, the Tg-albino fish can be raised to adulthood without any obvious defects (Fig. 6F,G and Supplementary Fig. S3C,D). Third, by using homogeneous substrates for the tRNA processing system in zebrafish, any unwanted complications that may possibly be caused by using heterogenous tRNAs will be excluded.

Studies of gene functions in zebrafish should be facilitated if a conditional mutagenesis system that allows generation of mutations in spatially and temporally regulated manners was established. Conditional CRISPR mutagenesis has been described by combining tissue-specific expression of Cas9 with ubiquitously expressed sgRNAs in transgenic fish^10–12^. In this study, we used an all-in-one construct containing both Cas9 and U6:gRNAs and demonstrated that the tRNA-based system is applicable to transgenic expression of multiple sgRNAs in zebrafish. With this system, cells over the whole body should have been mutagenized. We think the tRNA-gRNA system could further be applied to generate conditional mutants by constructing U6:tRNA-gRNAs lines with tissue-specific Cas9 lines and crossing them. The tissue-specific expression of Cas9 may be achieved by using a tissue-specific promoter as has been described^10–12^, or alternatively Cas9 may be expressed from a binary system, such as the Gal4-UAS system which will make hundreds of tissue-specific Gal4 lines available for this purpose^37^. Furthermore, more options for conditional mutagenesis will be generated if expression of sgRNAs can also be regulated. Whereas the Pol III-dependent U6 promoter induces ubiquitous expression of tRNA-gRNAs, the use of Pol II promoters enables tRNA-gRNAs expression in temporally and spatially restricted fashions. It is expected that such temporally- and/or spatially-regulated sgRNA expression would improve the specificity of *in vivo* mutagenesis and could minimize accumulation of off-target mutations by limiting duration of mutagenesis. Therefore, the next important question to be solved is whether a transcript containing multiple tRNA-sgRNA fusions transcribed from an RNA polymerase II (Pol II) promoter can be processed properly and can generate functional sgRNAs in transgenic fish. Previously, it was shown that sgRNA surrounded by ribozymes, Hammerhead (HH) and hepatitis delta virus (HDV), placed downstream of the *hsp* promoter was functional when injected with Cas9 mRNA^19^. It has however not been shown if the pol II-mediated expression of both gRNAs and Cas9 can work in stable transgenic fish. Based on the development of our tRNA-based system described in this manuscript, works are in progress to develop the binary mutagenesis system involving UAS:Cas9 transgenic fish and transgenic fish carrying the Pol II-dependent tRNA-gRNA expression system.

Zebrafish is highly suited for *in vivo* imaging of biological phenomena at subcellular and cellular resolution owing to its transparency at early embryonic stages. However, at larval stages the optical accessibility is significantly limited by melanophores (black pigment), which is developed from ~24 hpf. Previous studies mainly reduced dark pigmentation by two different ways; (1) by inhibiting melanization of melanophores using chemical compounds 1-Phenyl-2-thiourea (PTU)^32^, or (2) by using pigmentation mutants that show defects in the development of melanophores, such as *nacre*, *casper* and *albino*^33–35^. The PTU treatment is a convenient and effective method, but is supposed to interfere not only with tyrosinase but also with various other enzymes, such as thyroid peroxidase^38^, and often causes some abnormalities in development and behaviors^35,38–40^. Unlike PTU-treated fish, mutants such as *nacre* and *albino* show normal development and behavior^33,35,40^, and in general more suitable for *in vivo* imaging analysis. A shortcoming of using mutants is that it requires at least one generation time (~3 months) to obtain a homozygous mutant line in the transgenic background of interest and time-consuming efforts to identify small numbers of homozygous larvae carrying the transgene. Therefore, the Tg-albino fish described in this study should be useful for *in vivo* imaging, especially for high-throughput screening of a huge number of transgenic resources, since transparent embryos and larvae can be obtained immediately after crossing any transgenic line of interest with the Tg-albino fish.

## Methods

### Ethics statement

This experiment was approved by the Institutional Animal Care and Use Committee (IACUC, approval identification number 27-2) at the National Institute of Genetics, and complied with the Guide for the Care and Use of Laboratory Animals of the IACUC.

### Zebrafish husbandry

Adult zebrafish were maintained at 25 °C under a regular 13 h light/11 h dark cycle, and fed twice per day. Embryos were kept at 28.5 °C under the same light/dark cycle until the larval stage.

### Plasmid DNA

Nine zebrafish tRNA genes, Dr-tRNA^Gly^(GCC), Dr-tRNA^Lys^(CTT), Dr-tRNA^Asn^(GTT), Dr-tRNA^Met^(CAT), Dr-tRNA^Gln^(CTG), Dr-tRNA^Ser^(GCT), Dr-tRNA^Thr^(AGT), Dr-tRNA^His^(GTG), and Dr-tRNA^Leu^(CAG), were artificially synthesized and cloned directly downstream of a modified sgRNA scaffold sequence^25^ (Fig. 1B) as follows: Dr-tRNA^Gly^(GCC) together with the sgRNA scaffold was inserted into the *Eco*RV site of pUC57. For cloning of other tRNA genes, a DNA fragment containing *Bsa*I site at the downstream end of the sgRNA scaffold was amplified by PCR, and cloned between the first *Nde*I site and the *Apa*I site of pUAS:Cas9T2AGFP;U6:sgRNA1;U6:sgRNA2 (Addgene plasmid 74009) to generate ptRNA-empty. Then, the other eight tRNA genes were inserted into the *Bsa*I site of the ptRNA-empty.

### Microinjection of Cas9 mRNA and sgRNAs

sgRNAs were designed using CRISPRscan (http://www.crisprscan.org/) and synthesized essentially as previously described^41^. Unless noted otherwise, PCR was performed using PrimeSTAR GXL DNA Polymerase (R050A, TaKaRa) following the manufacturer’s instructions. While the original sgRNA scaffold (sgTail primer1) was used for the validation of sgRNA efficiency (Fig. 2), a modified version of sgRNA scaffold^25^ (sgTail primer2) was used for subsequent experiments.

DNA templates for sgRNA synthesis were prepared from DNA oligos by fill-in PCR. DNA oligos used for the synthesis of sgRNAs are listed in Supplementary Table S2. DNA templates for the synthesis of sg1-tRNA^Gly^-3′ and sg1-HDV-3′ were amplified from tRNA plasmids by PCR. On the other hand, DNA templates for the synthesis of 5′-tRNA-sg1 and 5′-HH-sg1 were generated by two rounds of PCR as follows. First tRNA-sgRNA fragments consisting of tRNA and 20 bp-target sequences were amplified from tRNA plasmids by PCR, and then combined with the universal sgRNA scaffold fragment by overlap extension PCR. Primers used for the synthesis of each tRNA- or ribozyme-fused sgRNA are listed in Supplementary Table S3. sgRNAs, tRNA-fused sgRNAs, and ribozyme-fused sgRNAs were produced from the DNA templates by *in vitro* transcription using MEGAshortscript T7 Transcription Kit (AM1354, Ambion).

DNA templates for the synthesis of long tRNA-sgRNA fusion RNAs (3xalb-sgRNAs and 3xalb-3xmpv-sgRNA) were generated from the plasmid vectors for transgenesis (described below) by PCR using a primer pair (T7_Gly-GCC Fw and multi_sgRNA_IVT Rv; see Supplementary Table S3). These RNAs containing an array of tRNA-sgRNAs were synthesized by using MAXIscript T7 Transcription Kit (AM1312, Ambion).

Cas9 mRNA were transcribed from pCS2 + hSpCas9^42^ (Addgene plasmid 51815) by using the mMESSAGE mMACHINE SP6 Transcription Kit (AM1340, Ambion). 1–2 nL of mixture containing either 25 ng/µL sgRNA or 50 ng/µL tRNA-sgRNA, and 200 ng/µL Cas9 mRNA, 0.2 M KCl, and 0.05% phenol red was injected to 1-cell stage embryos. For the experiment of the long tRNA-sgRNAs, we injected a solution containing equimolar amounts of sgRNAs or tRNA-sgRNAs (10 ng/µL for sgRNA, 50 ng/µL for 3xalb-sgRNAs, or 100 ng/µL for 3xalb-3xmpv-sgRNA), and 200 ng/µL Cas9 mRNA, 0.2 M KCl, and 0.05% phenol red.

### Heteroduplex mobility assay

Heteroduplex mobility assay (HMA) was performed to detect mutations basically following the previous reports^29^. Genomic DNA was isolated from a mixture of three embryos at 24 hours-post-fertilization (hpf) or one larva at 120 hpf. A short fragment (100–200 bp) surrounding the target site was amplified from the genomic DNA by PCR using ExTaq (TaKaRa). The primers used are listed in Supplementary Table S4. The cycling conditions were as follows: one cycle at 94 °C for 2 min, followed by 35 or 40 cycles of 94 °C for 30 sec, 55 °C for 30 sec, and 72 °C for 30 sec. The resulting amplicons were analyzed by PAGE using SuperSep DNA gels (190–15481, Wako).

### RNA extraction and circularized reverse transcription-PCR (cRT-PCR)

Total RNA from ten embryos was extracted using TRIzol Reagent (15596026, Ambion). The procedure of cRT-PCR was illustrated in Supplementary Fig. S1 and was performed in accordance with the previous study^20^. To circularize RNA, 500 ng of total RNA was incubated in a 10 µL reaction mixture containing 1 x T4 RNA ligase buffer, 50 μM of ATP, 10% PEG8000, 10 U of RNaseOUT (Invitrogen) and 5 U of T4 RNA ligase 1 (M0204S, New England Biolabs). The ligation was carried out at 25 °C for 4 h. Then 2 U of Turbo DNase (Invitrogen) was added into the mixture to remove genomic DNA contamination at 25 °C for 30 min. The circularized RNA was purified with TRIzol Reagent and dissolved in nuclease free water. A total of 250 ng circularized RNA was reverse transcribed with slc45a2-sg1 RT Rv primer (5′-AACGT GCATA ATCAA CCTTC-3′) using SuperScript III First-Strand Synthesis System for RT-PCR (18080–051, Invitrogen) according to the manufacturer’s instructions. Then PCR was performed in 10 μL reaction containing 1/250 of cDNA using ExTaq (TaKaRa) with primers slc45a2-sg1 RT Rv primer (5′-AACGT GCATA ATCAA CCTTC-3′) and gRNA_RT-PCR Fw (5′-TGCTG GAAAC AGCAT AGCAA G-3′). The cycling conditions were as follows: one cycle at 94 °C for 2 min, followed by 25 cycles of 94 °C for 30 sec, 55 °C for 30 sec, and 72 °C for 15 sec. The resulting PCR products were analyzed with SuperSep DNA gels (190–15481, Wako). Subsequently, the cRT-PCR product was cloned into a pMD20-T vector using Mighty TA-cloning Kit (6028, TaKaRa). After plasmid purification, sequence analysis was carried out using the M13 forward primer and the M13 reverse primer.

### Generation of transgenic *albino* (Tg-albino) fish

The plasmid vector pT2TS-ubb:Cas9;u6c:Dr-tRNA^Gly^(GCC)-sgRNA-scaffold was used to express tRNA-sgRNA along with Cas9. The construct used for the generation of transgenic *albino* fish is illustrated in Fig. 6A. The pT2TS-ubb:Cas9;u6c:Dr-tRNA^Gly^(GCC)-sgRNA-scaffold consists of zebrafish *ubiquitin B* (*ubb*) promoter^31^ excised from pENTR5′_ubi (Addgene plasmid 27320), hSpCas9 derived from pCS2 + hSpCas9^42^ (Addgene plasmid 51815), poly-A addition signal, zebrafish U6c promoter^11^, zebrafish tRNA^Gly^(GCC), a cloning site (*Bsa*I and *Bse*RI) for multiple tRNA-sgRNAs, and a modified version of sgRNA-scaffold^25^. These DNA fragments were artificially synthesized unless otherwise stated and were cloned between the *Sal*I and *Hin*dIII restriction sites of pUAS:Cas9T2AGFP;U6:sgRNA1;U6:sgRNA2^12^ (Addgene plasmid 74009). The *ubb:Cas9* and *u6c:tRNA-sgRNA* are oriented in the opposite direction. tRNA-flanked sgRNAs are amplified from the zebrafish tRNA plasmids by PCR with primers listed in Supplementary Table S5, and cloned into the *Bse*RI site of the pT2TS-ubb:Cas9;u6c:Dr-tRNA^Gly^(GCC)-sgRNA-scaffold backbone to generate pT2TS-ubb:Cas9;u6c:3xalb-A, pT2TS-ubb:Cas9;u6c:3xalb-B pT2TS-ubb:Cas9;u6c:3xalb-C, and pT2TS—ubb:Cas9;u6c:3xalb-3xmpv (see Supplementary Fig. S6 for detailed cloning methods). The inserts and the backbone were assembled by using the In-Fusion HD Cloning Kit (639648, Clontech) following the manufacturer’s instructions. Assembling more than four fragments was inefficient as noted previously^21^. Therefore, to reduce the number of inserts to no more than three, we assembled two adjacent fragments into one longer fragment by overlap extension PCR as described previously^21^.

The Tg-albino fish, *Tg(ubb:SpCas9,u6c:3xslc45a2-sgRNA)*, was generated by injecting pT2TS-ubb:Cas9;u6c:3xalb-A with *tol2* transposase mRNA into fertilized eggs at the one-cell stage according to the previously study^43^. To identify transgenic founders, injected F0 adults were mated to wild-type. F1 progeny were firstly genotyped by PCR with a primer pair targeting SpCas9 (see Supplementary Table S4) at 1 dpf, and subsequently transgenic F1 progeny were screened by *albino*-like phenotype at 2 dpf.

### Imaging with a two-photon microscope

F1 Tg-albino fish was crossed with *Gt(gSA2AzGFF49A);Tg(UAS:GFP)*, and larvae carrying all the three transgenes were sorted under a fluorescence stereomicroscope (MZ16FA, Leica). For comparison, we prepared *Gt(gSA2AzGFF49A);Tg(UAS:GFP)* larvae in wild-type background or *nacre* background. Some of the larvae in wild-type background were maintained in E3 containing 0.003% 1-Phenyl-2-thiourea (PTU) from 24 hpf to inhibit pigmentation. The larvae were then mounted in 2% low-melting agarose at 5 dpf and observed with a fluorescence stereomicroscope (MZ16FA, Leica) equipped with a Leica DFC300 FX camera or with a two-photon microscope (LSM7MP, Carl Zeiss) to obtain EGFP fluorescence images. For two-photon imaging, the laser was tuned to 880 nm.

## Electronic supplementary material

Supplementary information

## Data Availability

The datasets generated during and analyzed during the current study are available from the corresponding author on reasonable request.
