# Supplementary material for: A tRNA-based multiplex sgRNA expression system in zebrafish and its application to generation of transgenic albino fish
Source: Sci Rep. 2018 Sep 6;8:13366. doi: 10.1038/s41598-018-31476-5 (PMC6127137; doi:10.1038/s41598-018-31476-5)
Supplement: Supplementary file 1 — Supplementary information [file 41598_2018_31476_MOESM1_ESM.pdf]

A tRNA-based multiplex sgRNA expression system in zebrafish and its application to generation of transgenic *albino* fish

Tomoya Shiraki and Koichi Kawakami

Supplementary information

Sequences of tRNA genes and ribozymes used in this study
tRNA 5' leader sequences are shown in lower-case
> Dr-tRNA^{Gly}(GCC)
gtgaGCATTGGTGGTTCAGTGGTAGAATTCTCGCCTGCCACGCGGGAGGCCCGGGTT
CGATTCCCGGCCAATGCA

> Dr-tRNA^{Lys}(CTT)
gttctcatcaGCCCGGCTAGCTCAGTCGGTAGAGCATGAGACTCTTAATCTCAGGGTCGTG
GGTTCGAGCCCCACGTCGGGCG

> Dr-tRNA^{Asn}(GTT)
gctatctGTCTCTGTGGCGCAATCGGTTAGCGCGTTCGGCTGTTAACCGAAAGGTTGGT
GGTTCGAGCCCACCCAGGGACG

> Dr-tRNA^{Met}(CAT)
gcctgaagGTTTCCGTAGTGTAGTGGTTATCACGTTCGCCTCATACGCGAAAGGTCCCCA
GTTCGAAACTGGGCGGAAACA

> Dr-tRNA^{Gln}(CTG)
gacttgaGGTTCCATGGTGTAATGGTTAGCACTCTGGACTCTGAATCCAGCGATCCGAGT
TCAAATCTCGGTGGGACCA

> Dr-tRNA^{Ser}(GCT)
ggaaaatGACGAGGTGGCCGAGTGGTTAAGGCGATGGACTGCTAATCCATTGTGCTTTG
CACGCATGGGTTCGAATCCCATCCTCGTCG

> Dr-tRNA^{Thr}(AGT)
gcagcGGCGCCGTGGCTTAGTTGGTTAAAGCGCCTGTCTAGTAAACAGGAGATCCTGG
GTTCGAATCCCAGCGGTGCCT

> Dr-tRNA^{His}(GTG)
gctcGCCGTGATCGTACAGTGGTTAGTACTCTGCGTTGTGGCCGCAGCAACCCCGGTT
CGAATCCGGGTCACGGCA

> Dr-tRNA^{Leu}(CAG)
gcatGTCAGGATGGCCGAGTGGTCTAAGGCGCTGCGTTCAGGTCGCAGTCTCCCCTG
GAGGCGTGGGTTCGAATCCCACTTCTGACA

>Os-tRNA^{Gly}(GCC)
gaacaaaGCACCAGTGGTCTAGTGGTAGAATAGTACCCTGCCACGGTACAGACCCGGG
TTCGATTCCCGGCTGGTGCA

>Os-tRNA^{Gly}(GCC)-scrambled
GAACCTCTTACACGCGCAGATCAACTAAATGTACACTGCGACGGTCCGTGGCTCCGA
GAGGGGTTACAGGGTACGCTG

>Dr-tRNA^{Gly}(GCC)-scrambled
GCGCTGTGGCGTACCGGGTACGTACTCGCTTGACTGGGTTGGTACTAGGCGAAACC
AGCTCCGTGGGATTGCACC

>Hammerhead ribozyme (HH)
gttccccCTGATGAGTCCGTGAGGACGAAACGAGTAAGCTCGTC

>Hepatitis delta virus ribozyme (HDV)
GGCCGGCATGGTCCCAGCCTCCTCGCTGGCGCCGGCTGGGCAACATGCTTCGGCAT
GGCGAATGGGAC

Supplementary Table S1.
Summary of zebrafish tRNA genes used in this study

tRNA Isotype	Anticodon	Gene number	tRNA # used in this study	tRNA-Score
Asn	GTT	1138	Chr15.trna38	82.89
Lys	CTT	1018	Chr5.trna677	81.83
Gly	GCC	826	Chr22.trna999	81.62
Met	CAT	612	Zv9-scaffold3503.trna115	84.46
Ser	GCT	436	Chr4.trna543	86.48
His	GTG	405	Chr4.trna5002	67.62
Thr	AGT	384	Chr7.trna183	84.06
Gln	CTG	297	Chr4.trna512	75.59
Leu	CAG	314	Chr22.trna605	78.35

Supplementary Table S2.
DNA oligos used for sgRNA synthesis

sgRNA Name	Sequence
drslc45a2_sg1	taatacgactcactataGGGGAAGGTTGATTATGCACgttttagagctagaa
drslc45a2_sg2	taatacgactcactataGGGGTCGGCCATGTTTGGAAgttttagagctagaa
drslc45a2_sg3	taatacgactcactataGGAGGTCGTCATGGGGCCGAgttttagagctagaa
drslc45a2_sg4	taatacgactcactataGGGGGTTGAAGTGGGCTGTTgttttagagctagaa
drslc45a2_sg5	taatacgactcactataGGGAGATGGAGTGGCTCCTGgttttagagctagaa
drmpv17_sg1	taatacgactcactataGGGGTCCAGTGGTCGGTGGAgttttagagctagaa
drmpv17_sg2	taatacgactcactataGGTGGTCGGTGGATGGTACAgttttagagctagaa
drmpv17_sg3	taatacgactcactataGGTTGTGATTGGCCAGTCCAgttttagagctagaa
drmpv17_sg4	taatacgactcactataGGAATAACTGGAACCCTCAAgttttagagctagaa
drmpv17_sg5	taatacgactcactataGGGAGGGATGAAATAAAAATgttttagagctagaa
sgTail primer1	aaaagcaccgactcggtgccactttttcaagttgataacggactagccttattttaacttgctatttctagctctaaaac
drslc45a2_sg1-2 (for sgTail primer2)	taatacgactcactataGGGGAAGGTTGATTATGCACgtttaagagctatgc
sgTail primer2	aaaagcaccgactcggtgccactttttcaagttgataacggactagccttatttaaacttgctatgctgtttccagcatagctcttaaac

Targeting sequences of sgRNAs are shown in upper-case

Supplementary Table S3.
DNA oligos used for the synthesis of 5’-tRNA-sgRNA, sgRNA-tRNA-3’, 5’-HH-sgRNA, and sgRNA-HDV-3’

sgRNA Name	Primer Name	Sequence
5’-Dr-tRNA^{Gly}(GCC)-sgRNA	T7_Gly-GCC Fw	taatacgactcactataGGTGAGCATTGGTGGTTCAGTG
	slc45a2-sg1_Gly−GCC Rv	GTGCATAATCAACCTTCCCCTGCATTGGCCGGGAATCGAAC
5’-Dr-tRNA^{Gly}(GCC)scr-sgRNA	T7-DrGly-GCC-sh Fw	taatacgactcactataGGCGCTGTGGCGTACCGGG
	slc45a2-sg1-DrGly-GCC-scr Rv	GTGCATAATCAACCTTCCCCGGTGCAATCCCACGGAGCTG
5’-Os-tRNA^{Gly}(GCC)-sgRNA	T7-OsGly-GCC Fw	taatacgactcactataGGAACAAAGCACCAGTGGTCT
	slc45a2-sg1-OsGly-GCC Rv	GTGCATAATCAACCTTCCCCTGCACCAGCCGGGAATCGAA
5’-Os-tRNA^{Gly}(GCC)scr-sgRNA	T7-OsGly-GCC-sh Fw	taatacgactcactataGGAACCTCTTACACGCGCAGAT
	slc45a2-sg1-OsGly-GCC-scr Rv	GTGCATAATCAACCTTCCCCCAGCGTACCCTGTAACCCCTCT
5’-HH-sgRNA	T7-HH Fw	taatacgactcactataGGTTCCCCCTGATGAGTCCGT
	slc45a2-sg1-HH Rv	GTGCATAATCAACCTTCCCCGACGAGCTTACTCGTTTC
5’-Dr-tRNA^{Asn}(GTT)-sgRNA	T7_Asn-GTT Fw	taatacgactcactataGGCTATCTGTCTCTGTGGC
	slc45a2-sg1_Asn-GTT Rv	GTGCATAATCAACCTTCCCCCGTCCCTGGGTGGGCTCGAA
5’-Dr-tRNA ^{Lys}(CTT)-sgRNA	T7_Lys-CTT Fw	taatacgactcactataGGTTCTCATCAGCCCGGCTA
	slc45a2-sg1_Lys-CTT Rv	GTGCATAATCAACCTTCCCCCGCCCGACGTGGGGCTCGAA
5’-Dr-tRNA ^{Met}(CAT)-sgRNA	T7_Met-CAT Fw	taatacgactcactataGGCCTGAAGGTTTCCGTAGTG
	slc45a2-sg1_Met-CAT Rv	GTGCATAATCAACCTTCCCCTGTTTCCGCCCAGTTTCG
5’-Dr-tRNA ^{Ser}(GCT)-sgRNA	T7_Ser-GCT Fw	taatacgactcactataGGAAAATGACGAGGTGGCCGA
	slc45a2-sg1_Ser-GCT Rv	GTGCATAATCAACCTTCCCCCGACGAGGATGGGATTCGAAC
5’-Dr-tRNA ^{His}(GTG)-sgRNA	T7_His-GTG Fw	taatacgactcactataGGCTCGCCGTGATCGTACAGT
	slc45a2-sg1_His-GTG Rv	GTGCATAATCAACCTTCCCCTGCCGTGACCCGGATTCGAAC
5’-Dr-tRNA ^{Thr}(AGT)-sgRNA	T7_Thr-AGT Fw	taatacgactcactataGGCAGCGGCGCCGTGGCTTA
	slc45a2-sg1_Thr-AGT Rv	GTGCATAATCAACCTTCCCCAGGCACCGCTGGGATTCGAAC
5’-Dr-tRNA ^{Gln}(CTG)-sgRNA	T7_Gln-CTG Fw	taatacgactcactataGGACTTGAGGTTCCATGGTGT
	slc45a2-sg1_Gln-CTG Rv	GTGCATAATCAACCTTCCCCTGGTCCCACCGAGATTTGAA
5’-Dr-tRNA ^{Leu}(CAG)-sgRNA	T7_Leu-CAG Fw	taatacgactcactataGGCATGTCAGGATGGCCGAGT
	slc45a2-sg1_Leu-CAG Rv	GTGCATAATCAACCTTCCCCTGTCAGAAGTGGGATTCGAAC
universal sgRNA scaffold for 5’-tRNA-sgRNA	slc45a2-sg1_gRNA Fw	GGGGAAGGTTGATTATGCACGTTTAAGAGCTATGCTGGAAACA
	AAAA-gRNA Rv	AAAAGCACCGACTCGGTGCCACTTT
universal Fw primer for sgRNA-tRNA(ribozyme)-3’	T7-slc45a2-sg1-gRNA Fw	aatacgactcactataGGGGAAGGTTGATTATGCACGTTTAAGAGCTATGCTGGAAACA
sgRNA-Dr-tRNA^{Gly}(GCC)-3’	DrGly−GCC Rv	AAAATGCATTGGCCGGGAATCGAAC
sgRNA-Dr-tRNA^{Gly}(GCC)-scr-3’	DrGly-GCC-scr Rv	AAAAGGTGCAATCCCACGGAGCTG
sgRNA-Os-tRNA^{Gly}(GCC)-3’	OsGly-GCC Rv	AAAATGCACCAGCCGGGAATCGAA
sgRNA-Os-tRNA^{Gly}(GCC)-scr-3’	OsGly-GCC-scr Rv	AAAACAGCGTACCCTGTAACCCCTCT
sgRNA-HDV-3’	HDV Rv	AAAAGTCCCATTCGCCATGCCGAAG
3xalb-sgRNAs 3xalb-3xmpv-sgRNA	T7_Gly-GCC Fw	taatacgactcactataGGTGAGCATTGGTGGTTCAGTG
	multi_sgRNA_IVT Rv	GAGCTAGTTGGTACCATCGATA

T7 promoter sequence is shown in lower-case.
Each 5’-tRNA (ribozyme)-sgRNA fragment was amplified from a respective plasmid vector by PCR, and then combined with the universal sgRNA scaffold for 5’-tRNA-sgRNA by overlap extension PCR.

Supplementary Table S4.
Primers used for genotyping PCR

Target	Primer Name (in Fig.2)	Forward (5' to 3')	Reverse (5' to 3')	Amplicon Length (bp)
drslc45a2_sg1&sg2	F1 & R1	TGGACTCGGAGCTTAAAGAGG	AGCAACACTGGCGTGACAAAC	169
drslc45a2_sg3_#1 (Fig.2)	F2 & R2	AGTGTTGCTGAGCGTTGGAC	TGCCCACTAACATCAGAATCCC	176
drslc45a2_sg3_#2 (Fig.4-6)	F2b & R2b	ACAGTCTGGTGTGGCTCATAAG	CTGTTGTGACTGCATCTC	177
drslc45a2_sg4	F3 & R3	AAGGGGAATCCGTATGCTGAAC	AGCCAGAAAGAATGAGCAGTTG	160
drslc45a2_sg5	F4 & R4	CTTTCTGTCACAGGAGCAAAGG	TGAGCCAACTGAACCATACAGG	108
drmpv17_sg1&sg2	F1 & R1	TGGGCTTGTGTTCGTGATTG	TTGTGGCCCACCTGGTCAAC	196
drmpv17_sg3	F2 & R2	CAAACAGATGTCTGTCTCGGTC	TGCAAATGGAGCAGAATCAC	197
drmpv17_sg4_#1 (Fig.2)	F3 & R3	CTGCCGTTTATATCTCCACAGG	TCAGGCGTGTGATAGATTACCC	137
drmpv17_sg4_#2 (Fig.4)	F3 & R3b	CTGCCGTTTATATCTCCACAGG	AGTACATTTGTGCCATCACAGTC	172
drmpv17_sg5	F4 & R4	GTTTCATTTCTCCAGCTTTGGC	GCTGCAGTGGCATTAGCATTAG	138
SpCas9	–	TGCTGAACGCCAAGCTGATTAC	ACGAACTCGCTTTCCAGCTTAG	370

Supplementary Table S5.
Primers used for plasmid construction

Fragment Name	Primer Name	Sequence
3xalb-A-sgRNA_Fragment1 (template: Dr-tRNA^{Asn}(GTT))	slc45a2 sg1-Primer F1 BseRI	GATTCCCGGCCAATGCAGGGGAAGGTTGATTATGCACGTTTAAGAGCTATGCTGGAA
	slc45a2 sg3-Asn-Primer R1	TCGGCCCCATGACGACCTCCCGTCCCTGGGTGGGCTCGAA
3xalb-A--sgRNA_Fragment2 (template: Dr-tRNA^{Lys}(CTT))	slc45a2 sg3-Primer F2	GGAGGTCGTCATGGGGCCGAGTTTAAGAGCTATGCTGGAA
	slc45a2 sg4-Lys-Primer RL BseRI	CAGCATAGCTCTTAAACAACAGCCCACTTCAACCCCCCGCCCGACGTGGGGCTCGAA
3xalb-B-sgRNA_Fragment1 (template: Dr-tRNA^{Asn}(GTT))	slc45a2 sg1-Primer F1 BseRI	GATTCCCGGCCAATGCAGGGGAAGGTTGATTATGCACGTTTAAGAGCTATGCTGGAA
	slc45a2 sg4-Asn-Primer R1	AACAGCCCACTTCAACCCCCCGTCCCTGGGTGGGCTCGAA
3xalb-B--sgRNA_Fragment2 (template: Dr-tRNA^{Lys}(CTT))	slc45a2 sg4-Primer F2	GGGGGTTGAAGTGGGCTGTTGTTTAAGAGCTATGCTGGAA
	slc45a2 sg3-Lys-Primer RL BseRI	CAGCATAGCTCTTAAACTCGGCCCCATGACGACCTCCCGCCCGACGTGGGGCTCGAA
3xalb-C-sgRNA_Fragment1 (template: Dr-tRNA^{Lys}(CTT))	slc45a2 sg1-Primer F1 BseRI	GATTCCCGGCCAATGCAGGGGAAGGTTGATTATGCACGTTTAAGAGCTATGCTGGAA
	slc45a2 sg3-Lys-Primer R1	TCGGCCCCATGACGACCTCCCGCCCGACGTGGGGCTCGAA
3xalb-C--sgRNA_Fragment2 (template: Dr-tRNA^{Asn}(GTT))	slc45a2 sg3-Primer F2	GGAGGTCGTCATGGGGCCGAGTTTAAGAGCTATGCTGGAA
	slc45a2 sg4-Asn-Primer RL BseRI	CAGCATAGCTCTTAAACAACAGCCCACTTCAACCCCCCGTCCCTGGGTGGGCTCGAA
3xalb-3xmpv-sgRNA_Fragment1 (template: Dr-tRNA^{Asn}(GTT))	slc45a2 sg1-Primer F1 BseRI	GATTCCCGGCCAATGCAGGGGAAGGTTGATTATGCACGTTTAAGAGCTATGCTGGAA
	slc45a2 sg3-Primer R1	TCGGCCCCATGACGACCTCCCGTCCCTGGGTGGGCTCGAA
3xalb-3xmpv-sgRNA_Fragment2 (template: Dr-tRNA^{Met}(CAT))	slc45a2 sg3-Primer F2	GGAGGTCGTCATGGGGCCGAGTTTAAGAGCTATGCTGGAA
	slc45a2 sg4-Primer R2	AACAGCCCACTTCAACCCCCTGTTTCCGCCCAGTTTCG
3xalb-3xmpv-sgRNA_Fragment3 (template: Dr-tRNA^{ser}(GCT))	slc45a2 sg4-Primer F3	GGGGGTTGAAGTGGGCTGTTGTTTAAGAGCTATGCTGGAA
	mpv17 sg1-Primer R3	CACCGACCACTGGACCCCCGACGAGGATGGGATTCGAAC
3xalb-3xmpv-sgRNA_Fragment4 (template: Dr-tRNA^{His}(GTG))	mpv17 sg1-Primer F4	GTCCAGTGGTCGGTGGAGTTTAAGAGCTATGCTGGAA
	mpv17 sg3-Primer R4	GACTGGCCAATCACAACCTGCCGTGACCCGGATTCGAAC
3xalb-3xmpv-sgRNA_Fragment5 (template: Dr-tRNA^{Lys}(CTT))	mpv17 sg3-Primer F5	TGTGATTGGCCAGTCCAGTTTAAGAGCTATGCTGGAA
	mpv17 sg4-Primer RL BseRI	CAGCATAGCTCTTAAACTTGAGGGTTCCAGTTATTCCCGCCCGACGTGGGGCTCGAA

Supplementary figure legends

Fig. S1 Schematic diagram of cRT-PCR (Related to Figure 3D). The 5'-Dr-tRNA^{Gly}(GCC)-sg1 transcript or processed mature sgRNA was circularized by self-ligation with T4 RNA ligase, and then reverse-transcribed with a primer designed near the 5'-end of the sgRNA. Subsequently, the fragments originated from the unprocessed or processed sgRNA were amplified by a pair of specific primers to investigate the processing efficiency of the tRNA-sgRNA transcript.

Fig. S2 Related to Figure 3E. Sequence chromatographs of the mature sgRNA processed from the 5'-Dr-tRNA^{Gly}(GCC)-sg1 transcript. The white arrowhead indicates the presumptive processing site, and the black arrowheads indicate the actual cleavage sites.

Fig. S3 Related to Figure 6. Generation of transgenic albino (Tg-albino) zebrafish. (A) Representative images of the lateral views of 3-mpf F0 fish. (lower) Some fish show mosaic albino phenotype. (B) Representative images of the dorsal views (left) and lateral views (right) of 3-dpf F1 larvae derived from F0#11 (founder) fish. F1 larvae with normal pigmentation (top), mild *albino* phenotype (middle), and severe *albino* phenotype (bottom). (C) Representative images of the lateral views of adult wild-type (WT; upper) and transgenic (Tg; lower) fish. (D) Magnified images of the trunk region of the fish shown in D. Scale bars: 5 mm (A), 1 mm (B), 1 cm (C), 2 mm (D).

Fig. S4 Related to Figure 6. Presence of transgenes in F1 larvae with *albino* phenotypes from F0#11 (A) and F0#14 (B) fish. The transgenes are detected with SpCas9 primers (top), whereas the primer pair against the exon4 of *mpv17* (control; mpv17-F1 and mpv17-

R1) is used as an internal positive control primer pair (bottom). Full gels are shown in Supplementary Fig. S12.

Fig. S5 Related to Figure 7. Eye and body pigmentation in Tg-albino at Day 5. Representative bright field images of dorsal views of wild-type (WT), PTU-treated WT (WT+PTU), *albino*, and Tg-albino backgrounds. Scale bars: 1 mm.

Fig. S6 A detailed method for the one-step cloning of tRNA-sgRNAs into pT2TS-ubb:Cas9;u6c:Dr-tRNA^{Gly}(GCC)-sgRNA-scaffold.

Fig. S7 Full scanned gels for heteroduplex mobility assay shown in Figure 2. (A) Full scanned gels of Fig. 2B. (B) Full scanned gels of Fig. 2D.

Fig. S8 Full scanned gels shown in Figure 3. (A) Full scanned gels used for quantification in Fig. 3A. The two gel were processed in parallel. The left gel is the full scanned image of Fig. 3A. (B) Full scanned gels used for quantification in Fig. 3B. The two gel were processed in parallel. The left gel is the full scanned image of Fig. 3B. Lanes unrelated to this study on the right gel were removed. (C) Full scanned gels of Fig. 3C. (D) Full scanned image shown in Fig. 3D.

Fig. S9 Full scanned gels for heteroduplex mobility assay shown in Figure 4. (A) Full scanned gels shown in Fig. 4B and Fig. 4E. (B) Full scanned gels shown in Fig. 4C and Fig. 4G. (C) Full scanned gel shown in Fig. 4D. (D) Full scanned gel shown in Fig. 4F.

Fig. S10 Full scanned gels for heteroduplex mobility assay shown in Figure 5. (A) Full scanned gel shown in Fig. 5B. (B) Full scanned gel shown in Fig. 5C. (C) Full scanned gel shown in Fig. 5D.

Fig. S11 Full scanned gels shown in Figure 6E.

Fig. S12 Full scanned gels shown in Supplementary Figure S4. (A) Full scanned gel shown in Supplementary Fig. S4A. (B) Full scanned gels shown in Supplementary Fig. S4B.

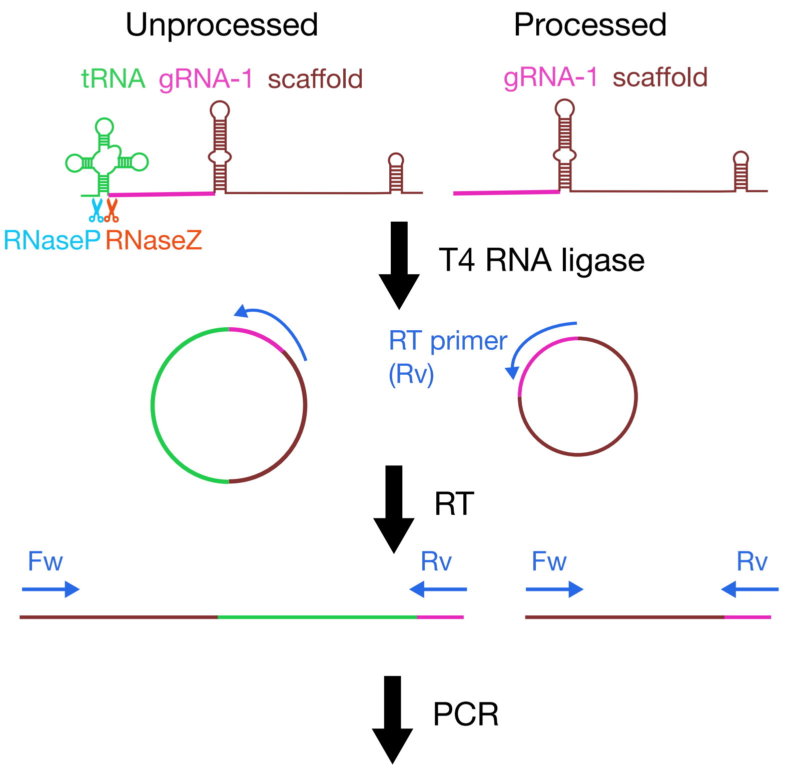
Unprocessed
Processed
tRNA
gRNA-1
scaffold
gRNA-1
scaffold
RNaseP
RNaseZ
T4 RNA ligase
RT primer
(Rv)
RT
Fw
Rv
Fw
Rv
PCR

Figure-S1

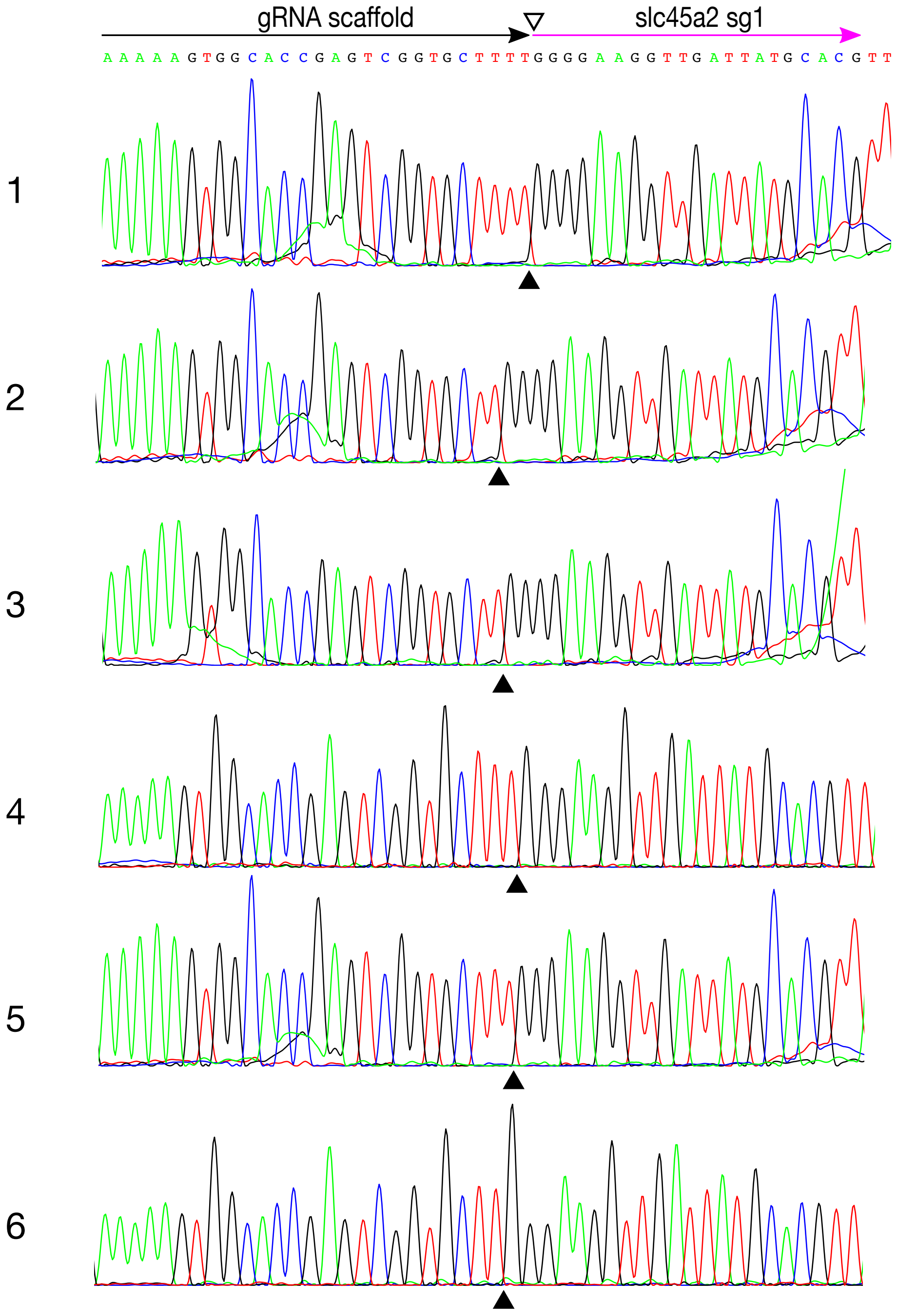
gRNA scaffold
slc45a2 sg1
A A A A A G T G G C A C C G A G T C G G T G C T T T T G G G G A A G G T T G A T T A T G C A C G T T
1
2
3
4
5
6

Figure-S2

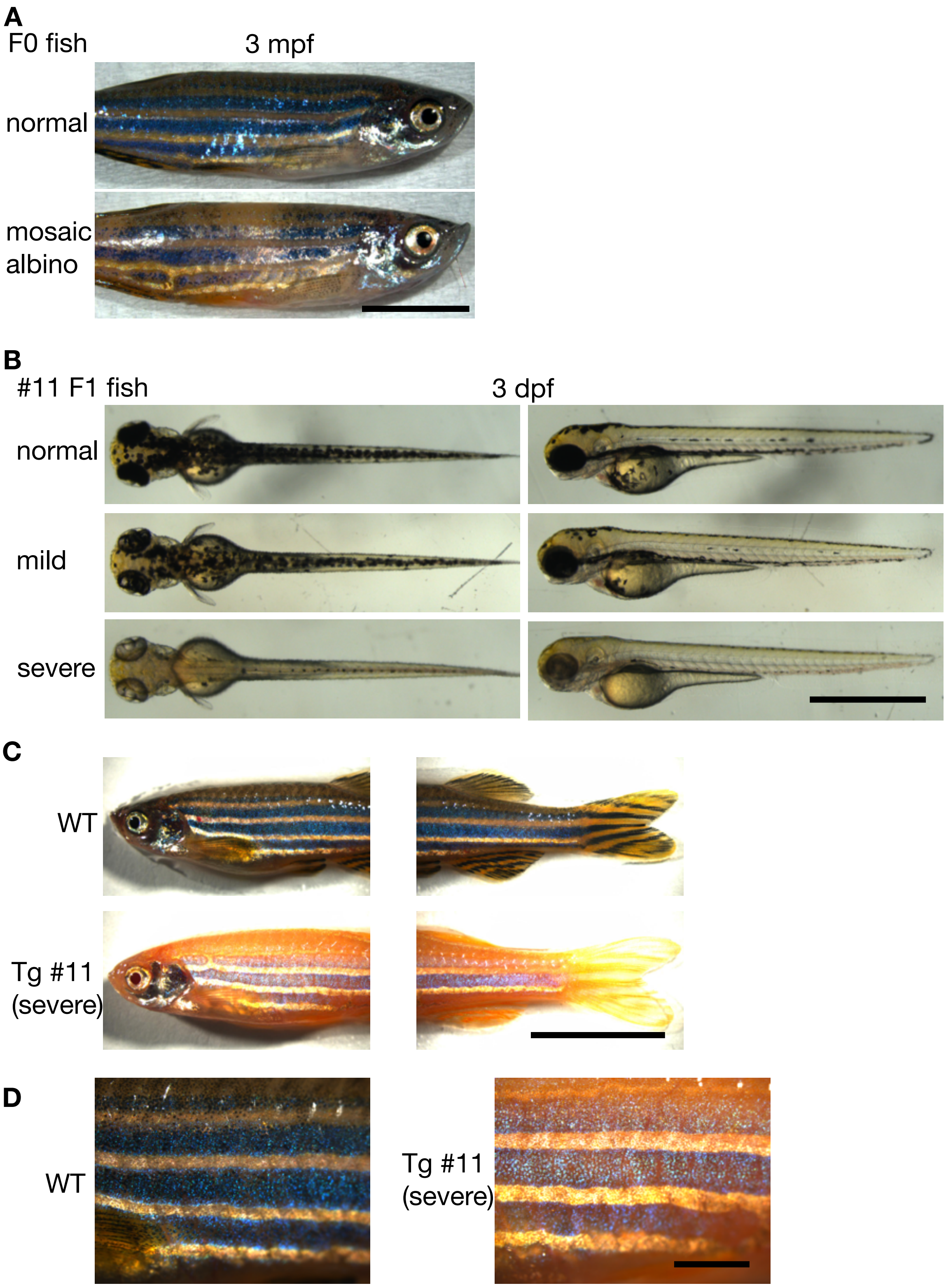
A
F0 fish
3 mpf
normal
mosaic albino
B
#11 F1 fish
3 dpf
normal
mild
severe
C
WT
Tg #11 (severe)
D
WT
Tg #11 (severe)

Figure-S3

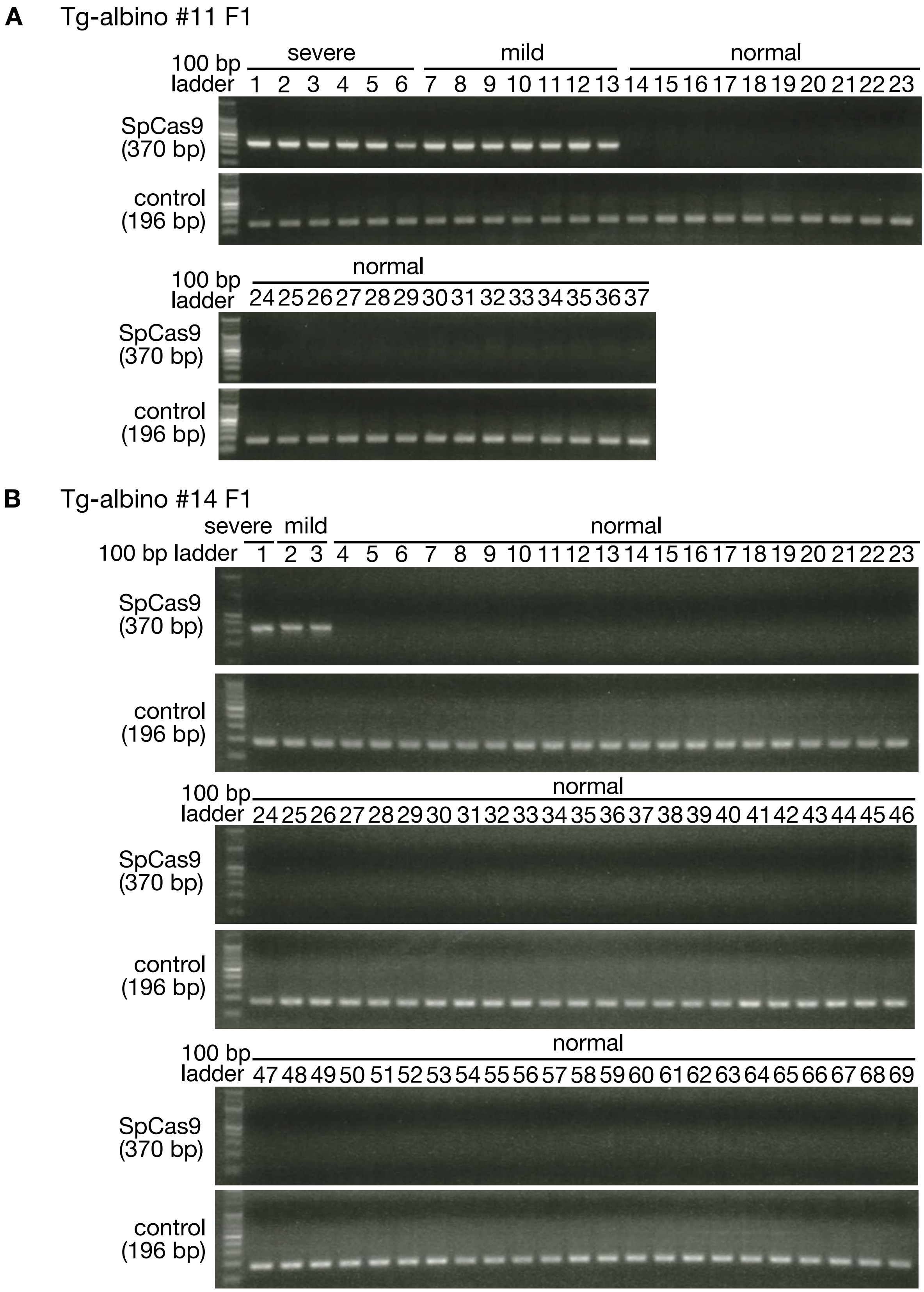
A
Tg-albino #11 F1
100 bp ladder
severe
mild
normal
1 2 3 4 5 6 7 8 9 10 11 12 13 14 15 16 17 18 19 20 21 22 23
SpCas9 (370 bp)
control (196 bp)
24 25 26 27 28 29 30 31 32 33 34 35 36 37
B
Tg-albino #14 F1
24 25 26 27 28 29 30 31 32 33 34 35 36 37 38 39 40 41 42 43 44 45 46
47 48 49 50 51 52 53 54 55 56 57 58 59 60 61 62 63 64 65 66 67 68 69

Figure-S4

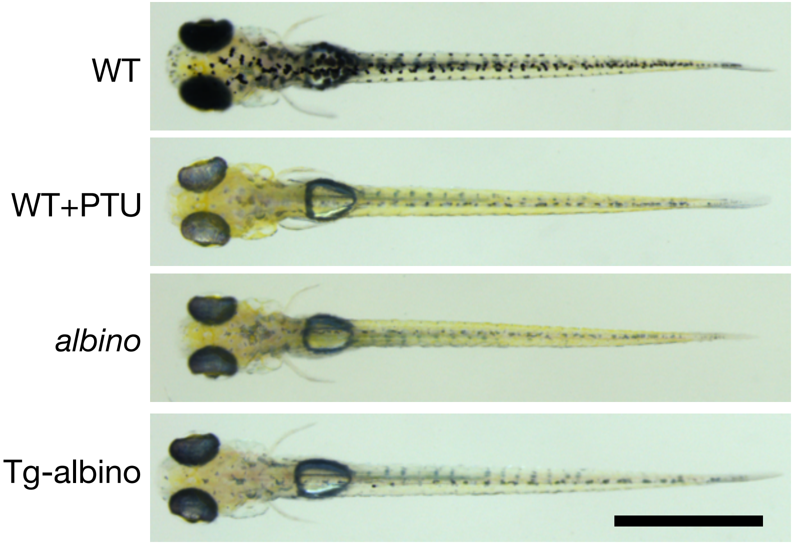

Figure-S5

1. Digest the pT2TS-ubb:Cas9;u6c:Dr-tRNA^{Gly}(GCC)-sgRNA-scaffold with *Bse*RI

(Only *Bse*RI is available due to the existence of *Bsa*I sites within the *ubb* promoter)

*Bse*RI produces cohesive ends that allow seamless cloning of sgRNA sequences.
(*Bse*RI cleavage sites are shown in orange, while *Bse*RI recognition sites are highlighted in yellow.)

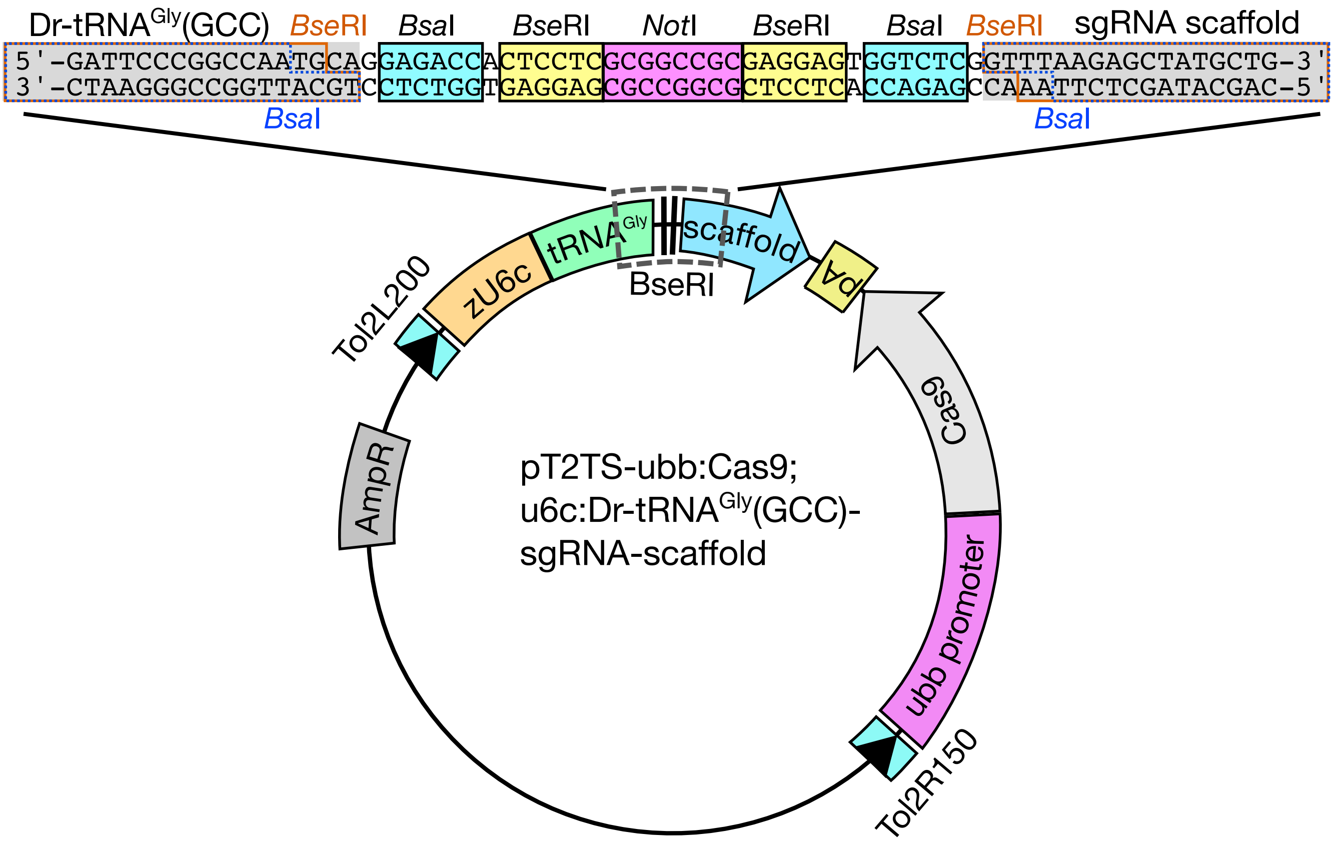

2. Prepare sgRNA fragments by PCR amplification

sgRNA fragments are amplified from pgRNA-drtRNA vectors by PCR, and then cloned into the linearized pT2TS-ubb:Cas9;u6c:Dr-tRNA^{Gly}(GCC)-sgRNA-scaffold by seamless cloning methods such as Gibson Assembly and In-Fusion as illustrated in the schematic below. The following schematic shows an example of the case of four sgRNAs.

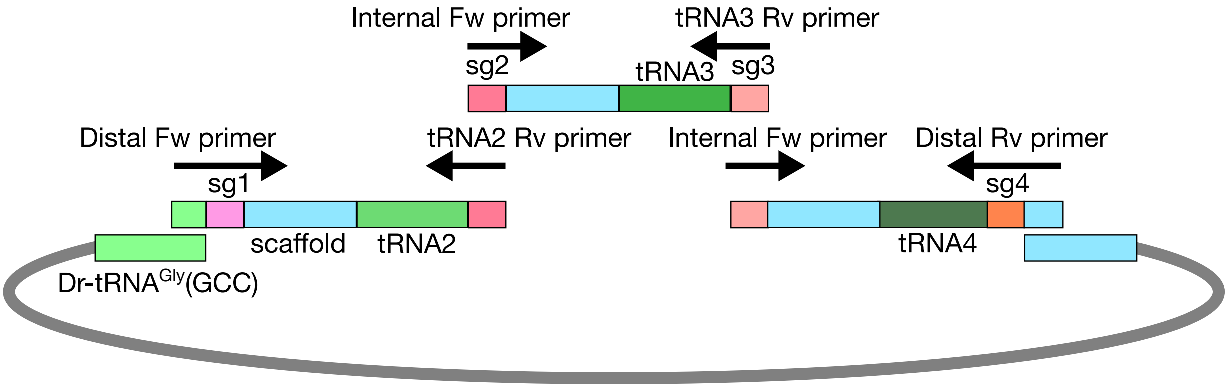

linearized pT2TS-ubb:Cas9;u6c:Dr-tRNA^{Gly}(GCC)-sgRNA-scaffold

Internal primers cotain ~20-nt sgRNA sequences used as homology arm for seamless ligation, while distal primers include 15-bp overlap with *Bse*RI-treated pT2TS-ubb:Cas9;u6c:Dr-tRNA^{Gly}(GCC)-sgRNA-scaffold.

Figure-S6

2-1. Design primers as shown in the table below

Primer Name	Primer Sequence	Template Vector
Distal Fw primer	tRNA^{Gly}(GCC) / sg1 guide sequence / sgRNA scaffold<br>GATTCCCGGCCAATGCANNNNNNNNNNNNNNNNNNNNGTTTAAGAGCTATGCTGGAA	
Internal Fw primer	sg guide sequence / sgRNA scaffold<br>NNNNNNNNNNNNNNNNNNNNGTTTAAGAGCTATGCTGGAA	
Asn Rv primer	sg guide sequence (rev comp) / tRNA^{Asn}(GTT)<br>NNNNNNNNNNNNNNNNNNNNCGTCCCTGGGTGGGCTCGAA	Dr-tRNA^{Asn}(GTT)
Met Rv primer	sg guide sequence (rev comp) / tRNA^{Met}(CAT)<br>NNNNNNNNNNNNNNNNNNNNTGTTTCCGCCCAGTTTCG	Dr-tRNA^{Met}(CAT)
Ser Rv primer	sg guide sequence (rev comp) / tRNA^{Ser}(GCT)<br>NNNNNNNNNNNNNNNNNNNNCGACGAGGATGGGATTCGAAC	Dr-tRNA^{Ser}(GCT)
His Rv primer	sg guide sequence (rev comp) / tRNA^{His}(GTG)<br>NNNNNNNNNNNNNNNNNNNNTGCCGTGACCCGGATTCGAAC	Dr-tRNA^{His}(GTG)
Thr Rv primer	sg guide sequence (rev comp) / tRNA^{Thr}(AGT)<br>NNNNNNNNNNNNNNNNNNNNAGGCACCGCTGGGATTCGAAC	Dr-tRNA^{Thr}(AGT)
Gln Rv primer	sg guide sequence (rev comp) / tRNA^{Gln}(CTG))<br>NNNNNNNNNNNNNNNNNNNNTGGTCCCACCGAGATTTGAA	Dr-tRNA^{Gln}(CTG)
Leu Rv primer	sg guide sequence (rev comp) / tRNA^{Leu}(CAG)<br>NNNNNNNNNNNNNNNNNNNNTGTCAGAAGTGGGATTCGAAC	Dr-tRNA^{Leu}(CAG)
Lys Rv primer	sg guide sequence (rev comp) / tRNA^{Lys}(CTT)<br>NNNNNNNNNNNNNNNNNNNNCGCCCGACGTGGGGCTCGAA	Dr-tRNA^{Lys}(CTT)
Distal Rv primer	gRNA scaffold / sg(last) guide sequence (rev comp) / tRNA X<br>CAGCATAGCTCTTAAACNNNNNNNNNNNNNNNNNNNNNNNNNNNNNNNNNNNNNNNN	Dr-tRNA X

2-2. Run PCRs

Run the individual PCRs using a high-fidelity polymerase according to the manufacturer's instructions. The protocol shown below is an example of the use of PrimeSTAR GXL (TaKaRa).

Set up the 1st PCR mixture

	final conc.
PrimeSTAR GXL Buf.	1x
dNTPs	0.2 mM each
Primer	0.3 μM each
Template Plasmid	1 ng/nL
PrimeSTAR GXL	0.5 U/20 μL

Run PCR

98°C	1 min	
98°C	10 sec	20 cycles
55°C	30 sec	
68°C	20 sec	

→ Run the PCR products on 2% agarose gel. The expected sizes are ~200 bp. Purify the PCR products using a commercial kit.

For cloning more than four sgRNAs (three fragements), we fuse fragments by overlap extension PCR.

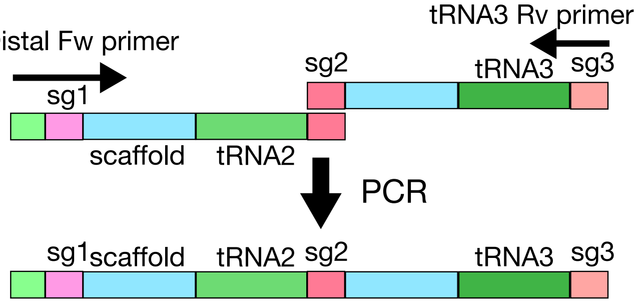

Set up the 2nd PCR mixture without primers (~100 ng each fragment in 20 μL reaction)

Run PCR without primers

98°C	1 min	
98°C	10 sec	15 cycles
55°C	30 sec	
68°C	20 sec	

Add primer pair (final conc., 0.3 μM each)

98°C	10 sec	20 cycles
55°C	30 sec	
68°C	30 sec	

2-3. Digest the PCR products with *Dpn*I to remove contaminating template plasmid DNA

Figure-S6
(continued)

3. Cloning sgRNA fragments

3-1. Seamless cloning using In-Fusion (or Gibson assembly)

A) Prepare the In-Fusion mixture

5x In-Fusion	0.5 μL
Vector backbone	~50 ng
Inserts (~200 bp)	~2 ng each
Inserts (~400 bp)	~4 ng each
Water	up to 2.5 μL

B) Incubate 50°C for 15 min

C) Transform the mixture into chemically competent cells, and plate cells on Ampicillin plates.

3-2. Select clones with correct inserts by colony PCR (This step can be skipped)

Perform colony PCR using primers below.

zU6c_seq Fw: 5'-TAAGCGTTTGCAGGTTTGCC-3'
h-globin-pA_seq Fw: 5'-AAAGGGAATGTGGGAGGTCAGTG-3'

3-3. Verify the recombinant colonies by Sanger sequencing with primers below

zU6c_seq Fw: 5'-TAAGCGTTTGCAGGTTTGCC-3'
h-globin-pA_seq Fw: 5'-AAAGGGAATGTGGGAGGTCAGTG-3'

Figure-S6
(continued)

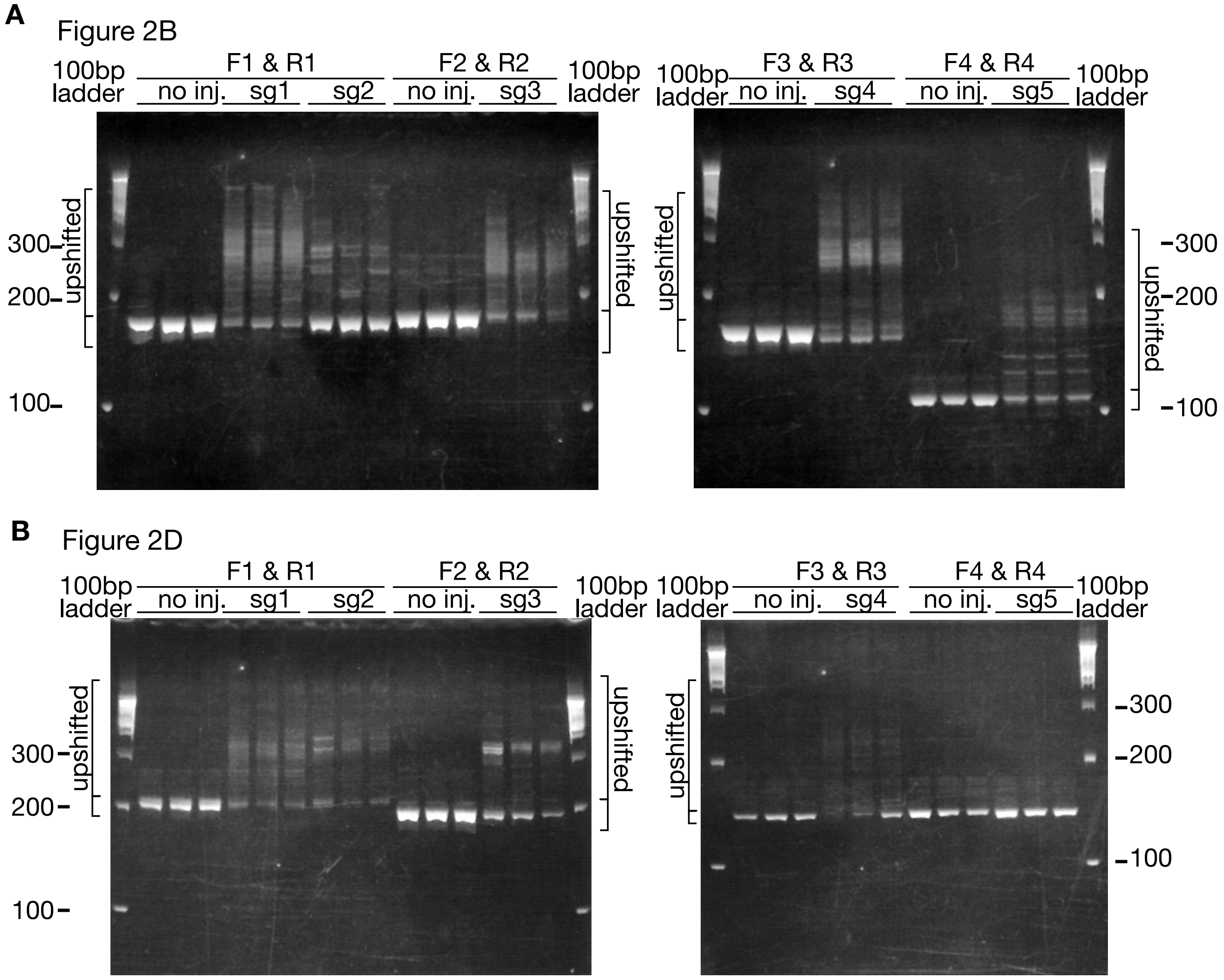

A
Figure 2B
100bp ladder
F1 & R1
no inj.
sg1
sg2
F2 & R2
no inj.
sg3
100bp ladder
100bp ladder
F3 & R3
no inj.
sg4
F4 & R4
no inj.
sg5
100bp ladder
upshifted
300
200
100
B
Figure 2D
100bp ladder
F1 & R1
no inj.
sg1
sg2
F2 & R2
no inj.
sg3
100bp ladder
100bp ladder
F3 & R3
no inj.
sg4
F4 & R4
no inj.
sg5
100bp ladder
upshifted
300
200
100

Figure-S7

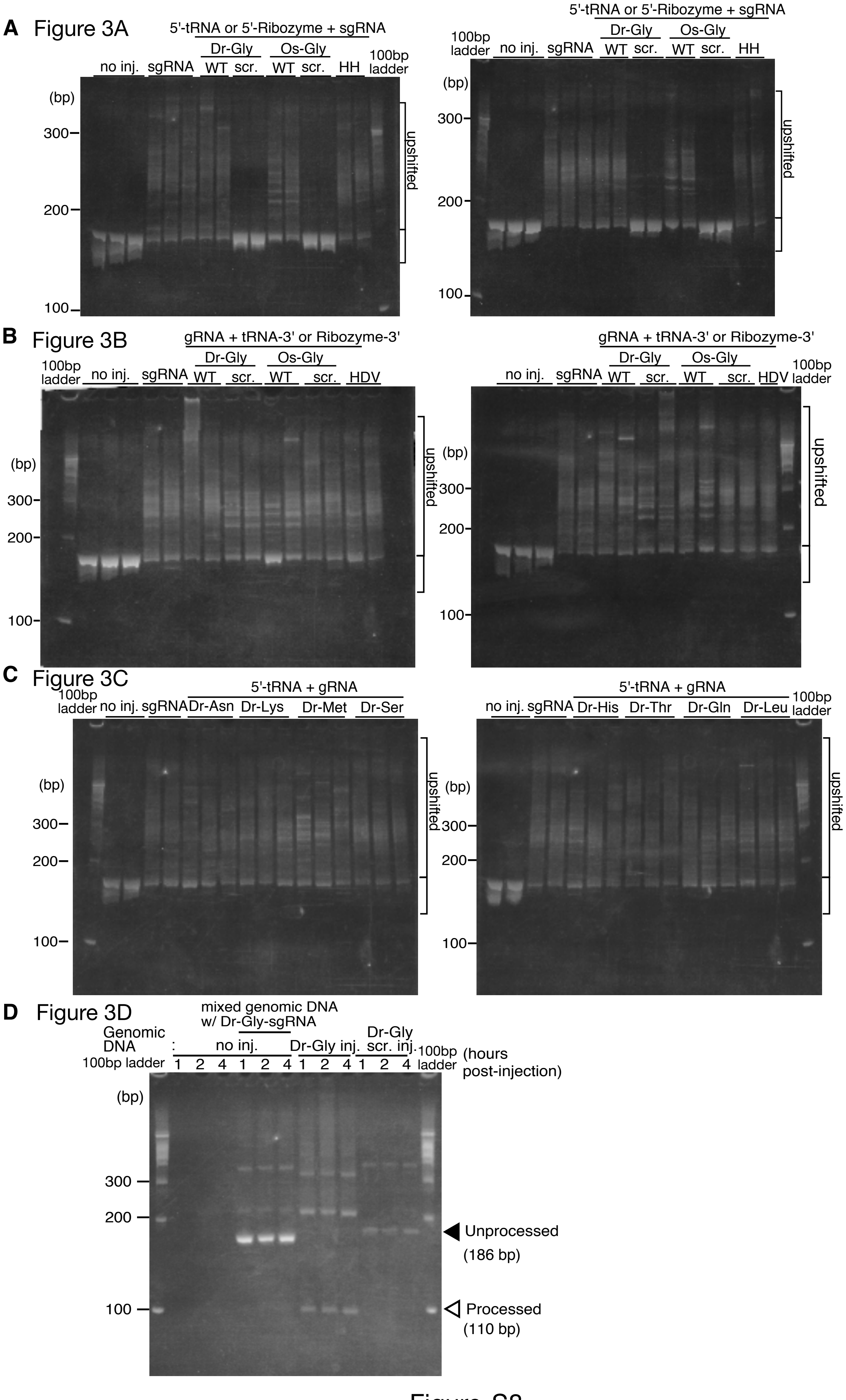
A Figure 3A
5'-tRNA or 5'-Ribozyme + sgRNA
Dr-Gly
Os-Gly
no inj.
sgRNA
WT
scr.
WT
scr.
HH
100bp ladder
(bp)
300
200
100
upshifted
100bp ladder
no inj.
sgRNA
Dr-Gly
Os-Gly
WT
scr.
WT
scr.
HH
5'-tRNA or 5'-Ribozyme + sgRNA
(bp)
300
200
100
upshifted
B Figure 3B
gRNA + tRNA-3' or Ribozyme-3'
Dr-Gly
Os-Gly
100bp ladder
no inj.
sgRNA
WT
scr.
WT
scr.
HDV
(bp)
300
200
100
upshifted
gRNA + tRNA-3' or Ribozyme-3'
Dr-Gly
Os-Gly
no inj.
sgRNA
WT
scr.
WT
scr.
HDV
100bp ladder
(bp)
300
200
100
upshifted
C Figure 3C
5'-tRNA + gRNA
100bp ladder
no inj.
sgRNA
Dr-Asn
Dr-Lys
Dr-Met
Dr-Ser
(bp)
300
200
100
upshifted
5'-tRNA + gRNA
no inj.
sgRNA
Dr-His
Dr-Thr
Dr-Gln
Dr-Leu
100bp ladder
(bp)
300
200
100
upshifted
D Figure 3D
mixed genomic DNA w/ Dr-Gly-sgRNA
Genomic DNA
100bp ladder
no inj.
Dr-Gly inj.
Dr-Gly scr. inj.
1 2 4 1 2 4 1 2 4 1 2 4
100bp ladder
(hours post-injection)
(bp)
300
200
100
Unprocessed (186 bp)
Processed (110 bp)

Figure-S8

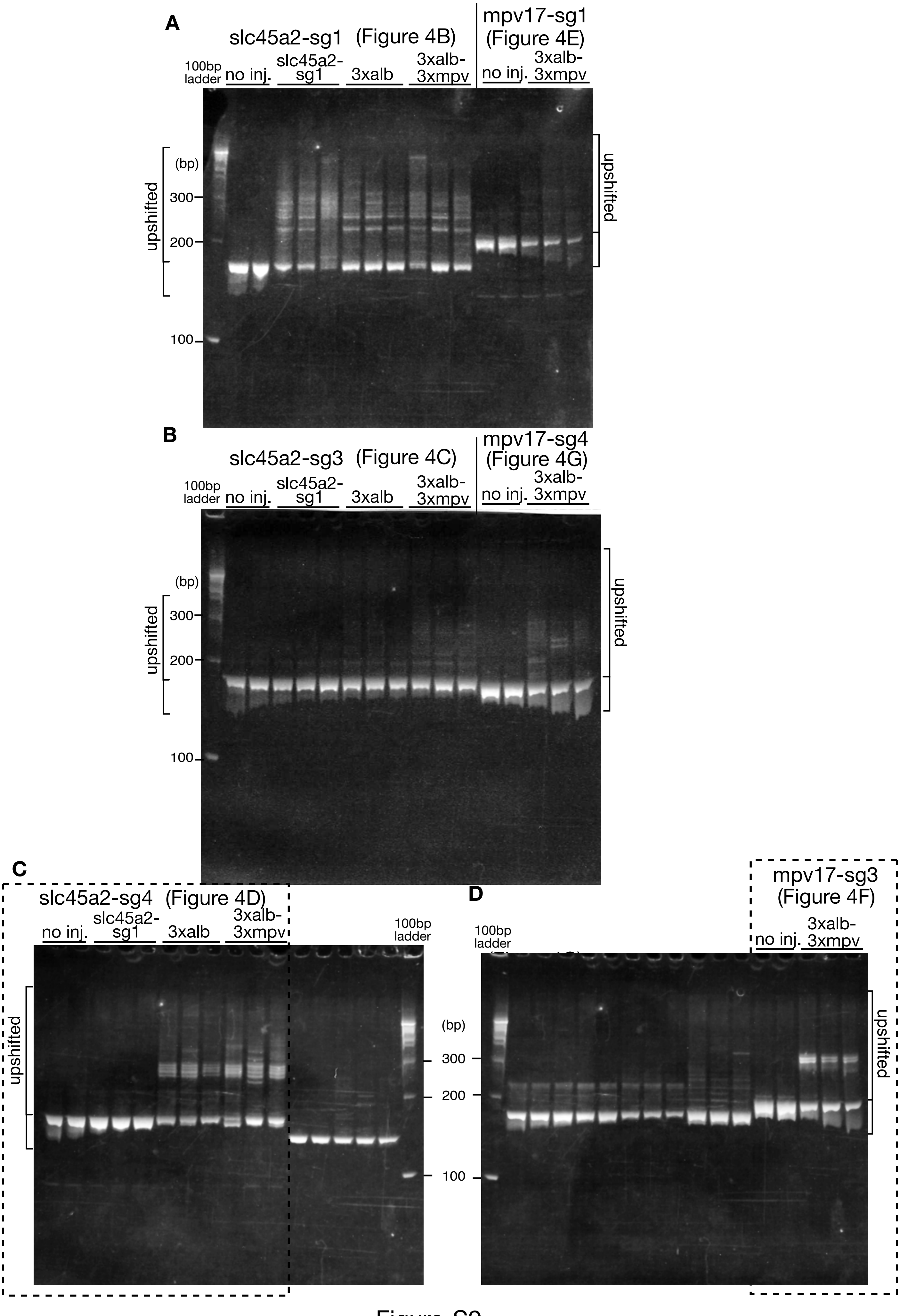
A
slc45a2-sg1 (Figure 4B)
mpv17-sg1 (Figure 4E)
100bp ladder
no inj.
slc45a2-sg1
3xalb
3xalb-3xmpv
no inj.
3xalb-3xmpv
(bp)
300
200
100
upshifted
upshifted
B
slc45a2-sg3 (Figure 4C)
mpv17-sg4 (Figure 4G)
100bp ladder
no inj.
slc45a2-sg1
3xalb
3xalb-3xmpv
no inj.
3xalb-3xmpv
(bp)
300
200
100
upshifted
upshifted
C
slc45a2-sg4 (Figure 4D)
no inj.
slc45a2-sg1
3xalb
3xalb-3xmpv
100bp ladder
(bp)
300
200
100
upshifted
D
mpv17-sg3 (Figure 4F)
100bp ladder
no inj.
3xalb-3xmpv
upshifted

Figure-S9

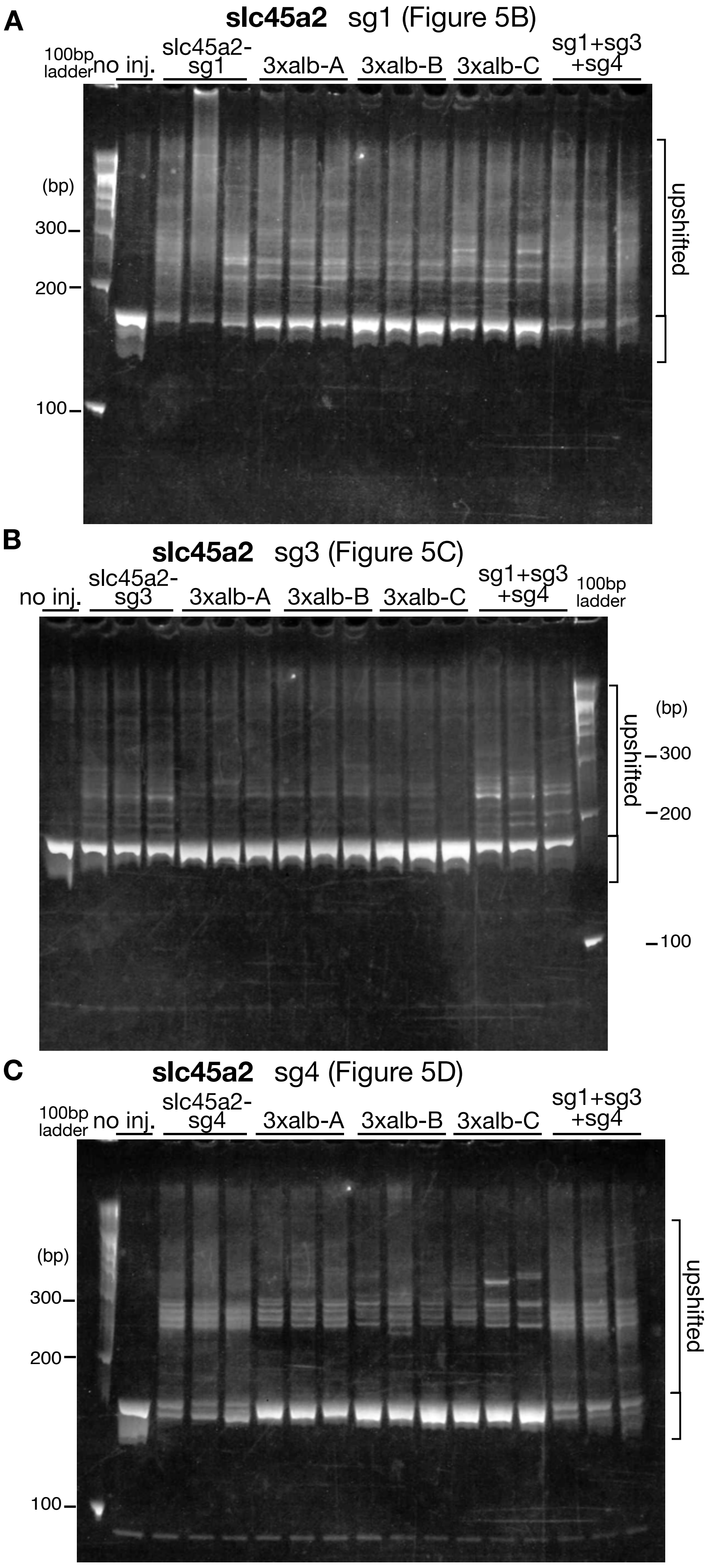
A
slc45a2 sg1 (Figure 5B)
100bp ladder
no inj.
slc45a2-sg1
3xalb-A
3xalb-B
3xalb-C
sg1+sg3 +sg4
(bp)
300
200
100
upshifted
B
slc45a2 sg3 (Figure 5C)
no inj.
slc45a2-sg3
3xalb-A
3xalb-B
3xalb-C
sg1+sg3 +sg4
100bp ladder
(bp)
300
200
100
upshifted
C
slc45a2 sg4 (Figure 5D)
100bp ladder
no inj.
slc45a2-sg4
3xalb-A
3xalb-B
3xalb-C
sg1+sg3 +sg4
(bp)
300
200
100
upshifted

Figure-S10

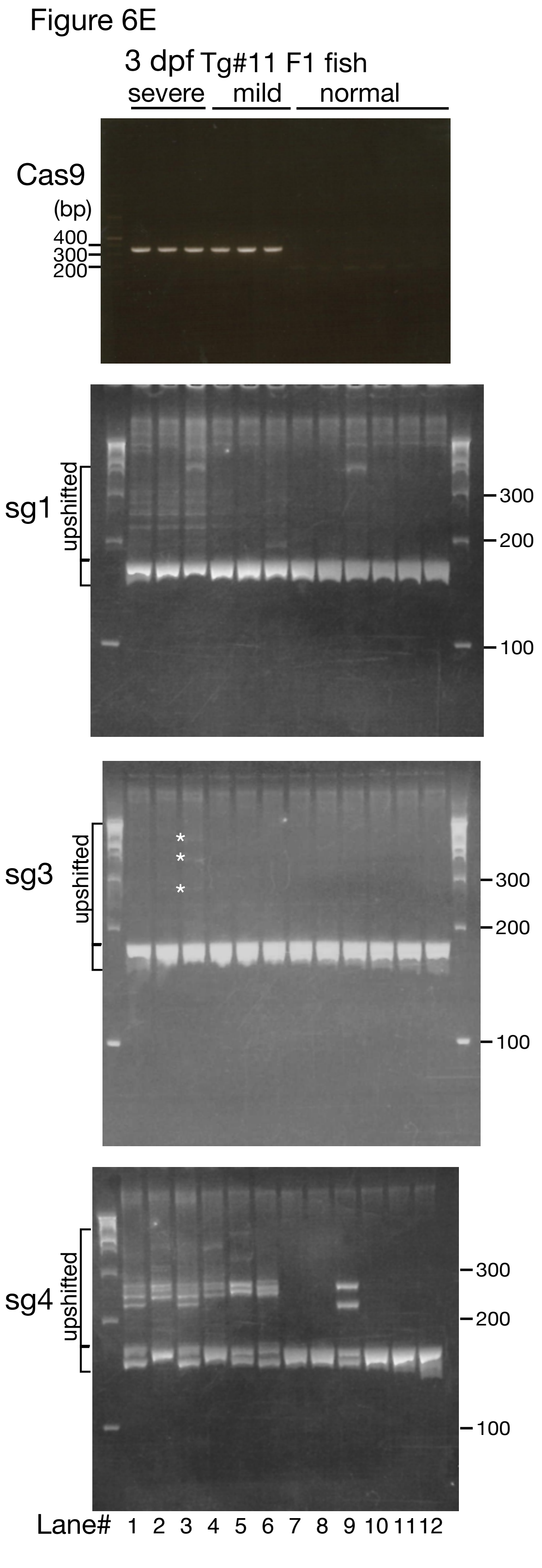
Figure 6E
3 dpf Tg#11 F1 fish
severe
mild
normal
Cas9
(bp)
400
300
200
sg1
upshifted
300
200
100
sg3
upshifted
*
*
*
300
200
100
sg4
upshifted
300
200
100
Lane# 1 2 3 4 5 6 7 8 9 10 11 12

Figure-S11

A Tg-albino #11 F1 (Figure S4A)

SpCas9
(370 bp)

control
(196 bp)

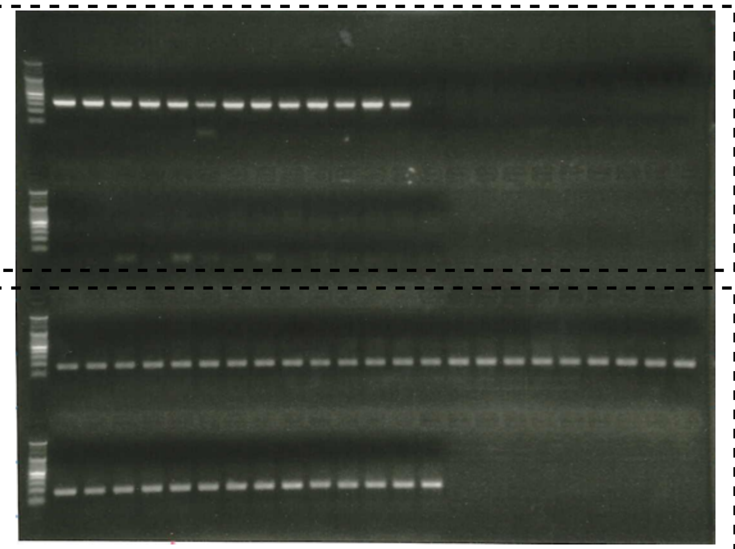

B Tg-albino #14 F1 (Figure S4B)

SpCas9
(370 bp)

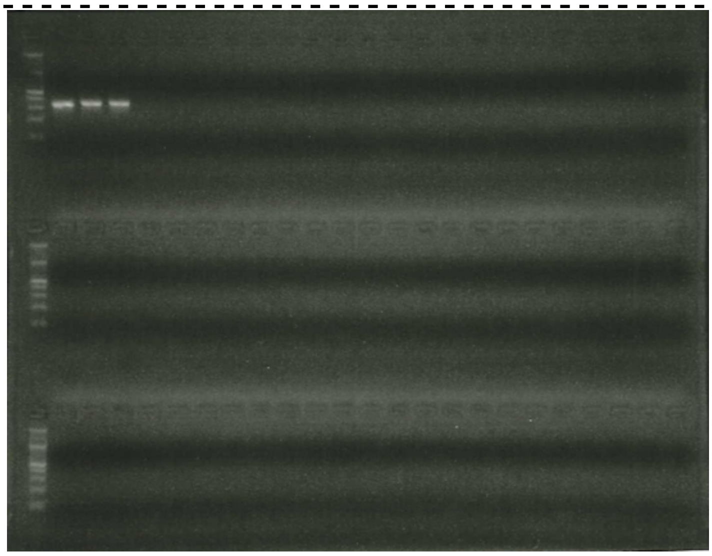

control
(196 bp)

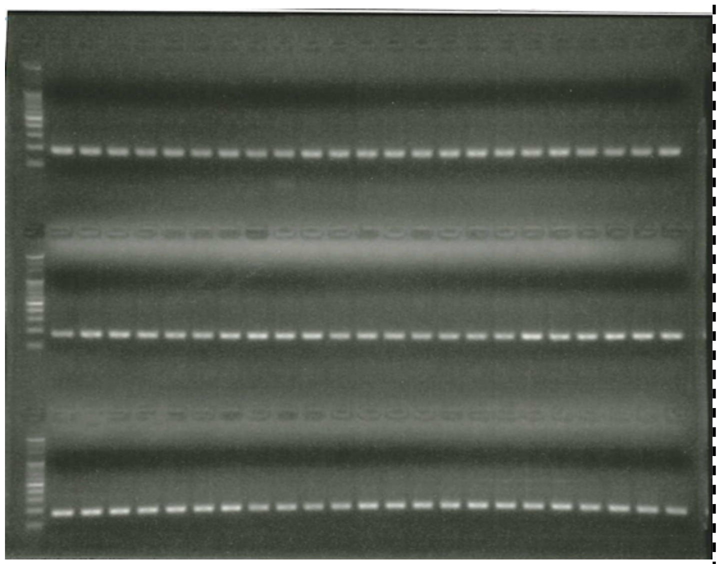

Figure-S12
